# A Mechanistic Understanding of Allosteric Immune Escape Pathways in the HIV-1 Envelope Glycoprotein

**DOI:** 10.1371/journal.pcbi.1003046

**Published:** 2013-05-16

**Authors:** Anurag Sethi, Jianhui Tian, Cynthia A. Derdeyn, Bette Korber, S. Gnanakaran

**Affiliations:** 1 Theoretical Biology and Biophysics, Los Alamos National Laboratory, Los Alamos, New Mexico, United States of America; 2 Center for Nonlinear Studies, Los Alamos National Laboratory, Los Alamos, New Mexico, United States of America; 3 Department of Pathology and Laboratory Medicine, Emory University, Atlanta, Georgia, United States of America; 4 Yerkes National Primate Research Center, Emory University, Atlanta, Georgia, United States of America; 5 Emory Vaccine Center, Emory University, Atlanta, Georgia, United States of America; 6 Center for HIV/AIDS Vaccine Immunology, School of Medicine, Duke University, Durham, North Carolina, United States of America; Harvard University, United States of America

## Abstract

The HIV-1 envelope (Env) spike, which consists of a compact, heterodimeric trimer of the glycoproteins gp120 and gp41, is the target of neutralizing antibodies. However, the high mutation rate of HIV-1 and plasticity of Env facilitates viral evasion from neutralizing antibodies through various mechanisms. Mutations that are distant from the antibody binding site can lead to escape, probably by changing the conformation or dynamics of Env; however, these changes are difficult to identify and define mechanistically. Here we describe a network analysis-based approach to identify potential allosteric immune evasion mechanisms using three known HIV-1 Env gp120 protein structures from two different clades, B and C. First, correlation and principal component analyses of molecular dynamics (MD) simulations identified a high degree of long-distance coupled motions that exist between functionally distant regions within the intrinsic dynamics of the gp120 core, supporting the presence of long-distance communication in the protein. Then, by integrating MD simulations with network theory, we identified the optimal and suboptimal communication pathways and modules within the gp120 core. The results unveil both strain-dependent and -independent characteristics of the communication pathways in gp120. We show that within the context of three structurally homologous gp120 cores, the optimal pathway for communication is sequence sensitive, i.e. a suboptimal pathway in one strain becomes the optimal pathway in another strain. Yet the identification of conserved elements within these communication pathways, termed inter-modular hotspots, could present a new opportunity for immunogen design, as this could be an additional mechanism that HIV-1 uses to shield vulnerable antibody targets in Env that induce neutralizing antibody breadth.

## Author Summary

The Env glycoproteins, gp120 and gp41, are the viral targets of HIV neutralizing antibodies. Accordingly, vaccine studies have focused on eliciting broadly neutralizing antibodies against epitopes in these proteins. Sequence diversity and the conformational flexibility of Env have made vaccine design efforts difficult. It is well documented that mutations distant from defined epitopes can lead to escape from neutralizing antibodies. In such cases, allostery within the Env protein could play a dominant role. In this study, we characterized the dynamical network in gp120 in terms of how spatially distant regions communicate with each other. We introduced an approach based on coupling computer simulations to compare gp120 core structures of three different virus strains from two clades, clade B and C. Our study finds that the long-distance collective motions in the protein are functionally relevant and are conserved across diverse strains of gp120, the communication pathways associated with these motions are sensitive to its sequence. Importantly, we find that gp120 exhibits communication modules (communities) with key residues (hotspots) serving as conduits for communication between different communities, a possible strategy to exploit in future vaccine design efforts.

## Introduction

The envelope (Env) glycoproteins, gp120 and gp41 are key vaccine components to induce antibody-mediated protection against HIV-1. Recently, monoclonal antibodies that can potently neutralize genetically diverse HIV-1 isolates have been recovered from a subset of HIV-1 infected individuals whose plasma exhibited exceptional neutralizing capacity [1]–[3]. All of these broadly neutralizing antibodies target conserved epitopes in either gp120 or gp41 to prevent viral entry into susceptible target cells. Furthermore, antibodies that bind to a conserved stretch of the gp120 variable loop (V1V2) domain conferred a modest level of protection against HIV-1 acquisition in the RV144 vaccine trial [4]. The humoral arm of the immune system is usually effective against viral infections and often contributes to complete clearance of a pathogen, resulting in the development of long-term immunity. However, in HIV-1, a delay in the induction of potent antibodies until well after the infection [5] has been seen along with viral evasion from neutralizing antibodies in natural infection through various mechanisms [6]–[9].

The extraordinary genetic diversity and the conformational plasticity of HIV-1 Env proteins, gp120 and gp41, present a formidable obstacle for effective immune control and vaccine design [3], [10], [11]. A rapid replication cycle, combined with the high error and recombination rates of the reverse transcriptase [12], [13] provide within-individual genetic diversity, which is then selected for immune evasion [6], [14]–[16]. Based on phylogenetic analysis, global HIV-1 sequences have been generally categorized into four groups (M, N, O and P), representing distinct introductions into humans, which can be further subdivided into clades and circulating recombinant forms [10], [17]–[19]. In addition, the clades tend to circulate in distinct geographical regions. The genetic diversity is driven by immune escape. When mutations occur within the antibody epitope, the mutations can directly reduce the binding affinity of the antibody to its target. In other cases, a mutation proximal to the epitope can change the glycosylation pattern of Env protein, creating a glycan shield that reduces accessibility of the epitope. Finally, escape mutations can occur in regions that are distal to the epitope [20]. These allosteric escape signatures take advantage of the conformational plasticity of Env proteins to evade antibody access to the epitope by changing the conformation or dynamics, and are thus much more difficult to identify and define mechanistically.

In a traditional sense, during allostery, a perturbation such as a mutation or ligand binding at an allosteric site induces a change in binding affinity of a second ligand at a distant active site. Allostery is often associated with a change in the conformation and/or dynamics of the protein [21], [22]. The energy landscape theory has been an effective tool to gain a mechanistic understanding of allostery. This theory states that a protein exists in more than one conformational state of comparable free energy in the absence of an allosteric effector [23], [24]. The binding of the allosteric effector changes the landscape and affects the relative populations of each of these states of the protein. The intrinsic dynamics of the protein cause conformational changes associated with differences in all of its regulatory states [23], [25]–[27]. In various neutralization studies of HIV-1, similar aspects of allostery have been observed. For example, antibodies may bind to known epitope (antibody-binding sites) in Env, but a spatially distant mutation alters the binding affinity of antibody and thereby leads to immune escape [20], [28]–[32].

In HIV-1, allosteric immune escape pathways are driven by the conformational plasticity of the Env subunits, gp120 and gp41, that associate in a trimeric fashion to form spikes in the viral membrane [33]. The Env subunits undergo large conformational changes following gp120 binding to receptors expressed by the target cell [34]–[39]. To enter a cell, gp120 initially binds to CD4, and then to a coreceptor (CCR5 or CXCR4), which are found together primarily on CD4 T cells [33], [38]–[40]. This viral entry process is highly allosteric in nature as gp120 binding to each receptor sequentially invokes a series of conformational changes. Thermodynamic studies indicate that significant changes in entropy are associated with the receptor binding process [35], [41]. The dynamics of the gp120 core by itself capture this inherent allostery [41], [42].

HIV-1 antibody neutralization profiles and associated allosteric immune escape often reflect sequence (or clade) specificity [8], [30], [32], [43]. It has been firmly established that clade B and C viruses exhibit different neutralization sensitivity and resistance patterns, even though only minor differences are seen in the three-dimensional structures of their gp120 proteins [44]. For example, bioinformatics analysis showed that certain residues in the α2 helix region of gp120 were under strong evolutionary pressure to evolve rapidly in clade C viruses, whereas this region was not under strong selective pressure in clade B viruses [30], [32]. Interestingly, only one neutralizing antibody epitope has been mapped exclusively to the α2 helix [45], [46], although residues in this region have contributed to other conformational epitopes [47], [48]. In addition, other studies suggest that there are immune escape mechanism(s) associated with α2 that have not been defined [30], [32]. Therefore, a mechanistic understanding of the conserved and variable features of allosteric communication in gp120, such as that exhibited by α2, could lead to the development of novel immunogen design strategies.

In the past, many different theoretical approaches have been employed to elucidate long-distance communication in proteins to obtain a mechanistic understanding of allostery. The lowest frequency normal modes of a simple elastic network have been successfully used to visualize the conformational changes associated with allostery [49]. However, normal modes are dependent on the structural fold of the protein and lack any sequence-specific effects. While the elastic network model assumes a harmonic approximation in the energy landscape of proteins, a quasi-harmonic analysis (also called Principal Component Analysis) of the fluctuations in the protein during equilibrium molecular dynamics (MD) simulations also identifies the coupled motion of a protein associated with allosteric conformational changes [50]–[54]. In addition, the dynamics of a protein in a MD simulation is sequence specific [55]–[58] and can be utilized to gain a molecular understanding of the conserved and variable features of allosteric communication in a protein family. Ranganathan and coworkers have used evolutionary analysis of proteins to show that a network of correlated residues plays a critical role in allosteric communication within several families of proteins [59], [60]. In spite of these studies, the exact role of sequence evolution in allosteric conformational pathways and the conservation of these pathways remain poorly understood.

In this study, we consider a topology-based analysis that utilizes network theory to capture the long-distance communication in gp120. Network analysis of macromolecules have been used in the past to identify the allosteric signaling pathways and conformational changes within proteins [27], [61]–[66]. These methods assume that energy transfer between local contacts leads to global communication in the macromolecule. The coupling in motion of residues in contact was used as a measure of information transfer between the two residues in the network as the motion in one residue can be used to predict the motion in the other residue [63], [67]. A number of communication pathways exist between an allosteric site and the active site in a protein, and these pathways can be identified using network theory. In the presence of multiple pathways for communication between distant sites, the role of each residue along the signaling pathway is queried. Recently, the modular nature of a network was studied in tRNA/protein complexes involved in translation [63]. This network was very dense within a module, while there were relatively fewer connections between different modules. It was shown that these inter-modular contacts were highly conserved by nature, and they existed in a majority of the suboptimal pathways within the macromolecule. This versatile method was then successfully applied to investigate the bottleneck for allosteric communication within metabolic protein-protein complexes [65], [68] and proteins involved in signaling [69]. However, the conservation of these modules in phylogenetically distant members of a similar structural fold has not been studied so far. As the dynamical network method utilizes information from MD simulations, it provides an ideal framework to investigate the conserved and variable aspects of the allosteric communication pathways in homologous proteins.

Here, we consider three different HIV-1 gp120 sequences. These gp120 proteins are chosen because their structures are highly conserved and are representative of the phylogenetic distances of HIV-1 Env sequences both within and between clades. The structures of each gp120 have been resolved in the CD4-bound conformation. Two structures are from clade B [70], [71] and the other is from clade C [44]. Clade B and C are the most prevalent phylogenetic forms of HIV-1 found in Europe/North America and Southern Africa respectively [17]. Historically, most studies of the structural aspects of Env gp120 to date have focused on clade B isolates, even though clade C alone accounts for more than 50% of global infections [72]–[74]. First, we employed a combination of correlation and principal component analyses to identify the dominant long-distance coupled motion between different functional regions in gp120 that are reminiscent of allosteric regulation. Then, network analysis was utilized to compare the communication pathways in gp120. We found that the shortest path for communication between distal regions is sensitive to the sequence of the individual protein; however, the modular structure of the allosteric network remains highly conserved. The inter-modular junctions (hotspots) form conduits for communication in the gp120 network and are associated with previously known antibody binding sites, and some of these residues are under high immune pressure to evolve rapidly. In addition, these hotspots have the potential to modify the dynamics of gp120 if a spatially proximal structural perturbation is introduced. Thus, we propose that reducing long-distance communication between distant regions of gp120 by appropriate choice of residues at the inter-modular junctions could be a viable strategy for structure-based immunogen design, as this approach would potentially expose vulnerable immune targets and control the conformational plasticity of the immunogen.

## Results

We performed long time scale unbiased all-atom molecular dynamics simulations of the gp120 structure from three different HIV-1 strains to characterize the communication network in these proteins and to identify the sensitivity of this network to its sequence. The HXB2 and YU2 strains both belong to clade B, while CAP210 belongs to clade C [75]. The high-resolution structure of the monomeric *apo* gp120 core (native monomer conformation without the CD4 ligand) remains unknown, although the structure of an HIV-1 gp120 homolog (SIV gp120) has been resolved in the absence of the CD4 receptor [76]. We find that the unliganded SIV structure does not lead to an optimal fit with the cryo-electron microscopy density maps of liganded and unliganded HIV-1 gp120 trimers on the viral membrane (Figure S1). This inconsistency was also noted in a previous study by Liu et al. [77]. Therefore, we carried out simulations of gp120 in the CD4-bound state and investigated the flexibility and dynamics of the protein in the absence of the CD4 receptor in these simulations. Additionally, we did not take into account the V1V2 hypervariable domain, as this region is expected to influence the gp120 core conformation [78] and the conformation of gp120 in the presence of the V1V2 domain remains unknown.

In all three simulations, gp120 protein did not undergo large conformational changes from the initial liganded metastable state during the timescale of our simulations (600 ns). This is consistent with the recent findings that the CD4-bound gp120 conformation is a reasonable approximation for the unliganded HIV-1 gp120 monomer core [78]. It was proposed that when the V1V2 domain and gp41 contacts are removed, the native gp120 core prefers a conformation that is similar to a CD4 bound conformation. A recent SAXS study demonstrated that when V1V2 is introduced onto a single monomer gp120 core, the core adopts a different conformation [41]. Therefore, by simulating a gp120 core without the V1/V2/V3 loops, we are considering a conformation that is representative of a preferred form of the gp120 core.

### Sequence independent long-distance coupled motions in gp120

Initially, we explored the intrinsic dynamics of gp120 for the long-distance strain independent collective motions in the three simulations of gp120 cores. Coupled motions between distant sites would be consistent with the presence of communication pathways within this protein. Since the sequence independent trends observed are similar across all three simulations, we present below the results only for the YU2 strain.

**Figure 1:**
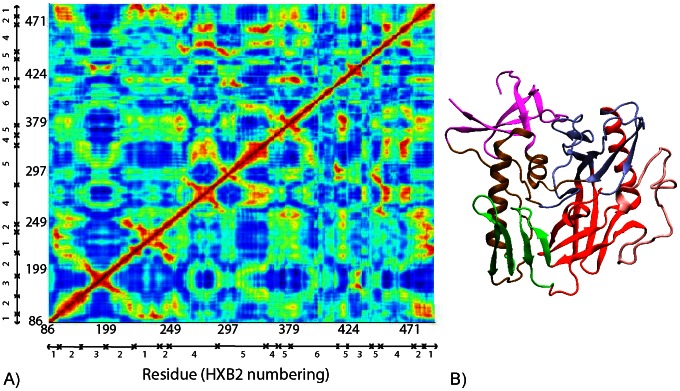
(A) The correlation of motion of each residue during the YU2 simulation. Blue represents anticorrelated motion, while red represents correlated motion. The residue numbering is HXB2. The subdomains are mapped in a gp120 core structure (B). Magenta represents the β-sandwich (region 1 in (A)), brown represents the αβ-bundle (region 2), green represents the bridging sheet (region 3), ice blue represents the 7-stranded barrel (region 4), red represents the 6-stranded barrel and α2 helix (region 5), and pink represents the V4 loop (region 6).

In Figure 1A, the degree of coupled motion between different residues in the YU2 simulation was measured by normalizing the covariance matrix of fluctuations in all Cα-atoms. Residues that move in the same direction in a coordinated fashion are correlated, while those that move in the opposite direction are anticorrelated. The inner domain of gp120 is composed of three subdomains: (i) a five-stranded β-sandwich, (ii) an αβ bundle containing two α-helices and two β-strands, and (iii) the bridging sheet (Figure 1B). The outer domain is also composed of three subdomains: (i) a barrel with seven strands, (ii) a six-stranded barrel along with the α2 helix, and (iii) the variable loop V4 (Figure 1B). Besides local correlations within each subdomain of the protein, the motions in distant regions of the protein are coupled. The β-sandwich, the αβ bundle, and the seven-stranded β-barrel are correlated in motion during the simulation. These three subdomains are anti-correlated in motion with the bridging sheet (that includes the stem of the V1/V2 loop), the six-stranded barrel, and the α2 helix in the outer domain. In turn, the motion of bridging sheet is correlated to the six-stranded barrel and the α2 helix of the outer domain.

We show that these long-distance coupled motions are similar across the three gp120 sequences (Table S1). The patterns in the covariance matrix are highly similar across all three MD simulations (Table S1). Also, the covariance of motion between residues converged during the timescale of each simulation (Text S1 and Figure S2). Most significantly, the motions of regions that undergo a conformational change following CD4 binding are coupled: the bridging sheet and the CD4-binding loop undergo large conformational changes upon CD4 binding, and the motions in these regions are anticorrelated, indicating that they move in a coordinated fashion.

### Sequence dependent and independent aspects of the dominant collective motions in gp120

Principal component analysis (PCA) was used to identify the dominant long-distance coupled motions in gp120. Generally, these long-distance coupled motions are associated with functional regulation [50]–[54], [79]. In PCA, quasi-harmonic analysis is carried out on time series trajectories from MD simulations to identify the coupled motions that dominate protein dynamics. Here, we expect the collective motions to be somewhat different in the three gp120s since these motions depend on the individual sequences, but the collective motions associated with CD4 and co-receptor binding should be conserved regardless of the strain because the entry process is invariant. Any differences in dominant collective motions of this protein can be informative of strain-specific features in communication pathways within the protein. We performed PCA on a single trajectory formed by merging 600 ns trajectories from all three gp120 simulations to analyze the differences and similarities in the long-distance coupled motion. Approximately 65% of the fluctuations of gp120 observed in the three simulations are along the first three PCs.

**Figure 2:**
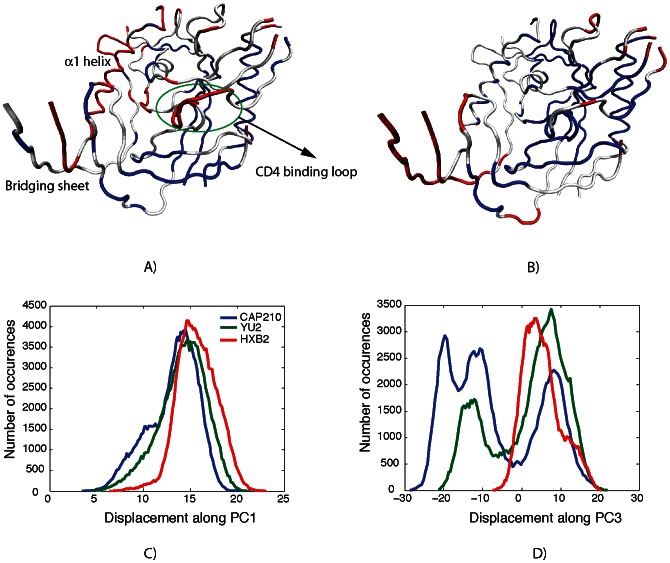
The coupling of motions along (A) PC1 and (B) PC3. Residues that are flexible in the corresponding PC are shown in red, while rigid regions are shown in blue. Regions that display moderate motion in the corresponding PC are shown in white. The histogram of displacement along (C) PC1 and (D) PC3 are also shown.

The fluctuations along PC1 (40%) and PC3 (10%) are nearly equivalent in all three simulations (Figure 2C&D), indicating that the motion in these PCs might pertain to the common functional dynamics of the gp120 family. In the PC1, the motion of distant regions in the protein that exhibit large conformational changes upon CD4 binding is coupled. More precisely, the bridging sheet, the CD4 binding loop, and the α1-helix exhibit large fluctuations in PC1 (Figure 2A, red). The CD4 binding loop interacts with CD4 in the CD4-bound complex, while the bridging sheet is formed only in CD4-receptor bound conformations or in gp120 core monomer without the V1 and V2 loops. Similar motions are also observed along PC3 in which the motion of parts of the bridging sheet is coupled to that of the inner domain and a small portion of the CD4 binding loop in the simulation (Figure 2B, red). Consistent with experimental measurements, these regions are highly flexible in all three simulations (Text S1 and Figures S3 to S5). As the motions of distant functional sites in all three sequences of gp120 are coupled in PC1 and PC3, the dominant coupled motions in gp120 are reminiscent of allosteric coupling. Similar long-distance coupled motions have been associated with allosteric conformational changes in many proteins [50]–[54], [79]. However, the role of individual principal components in the conformational changes associated with gp120 binding to CD4 receptor binding are difficult to evaluate, since the high-resolution structure of the monomeric *apo* gp120 core is unknown, and allostery in the gp120 core may be entropic in nature.

**Figure 3:**
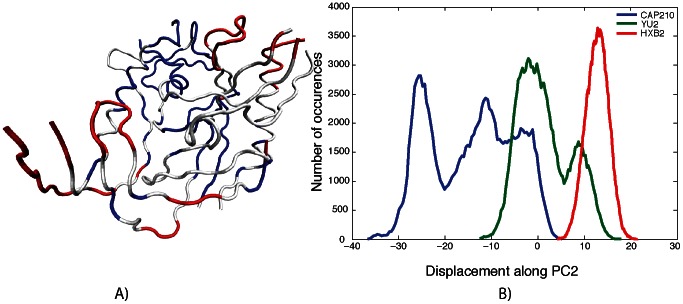
The coupling of motion along PC2 (A). Colors are as defined in Figure 4. The histogram of displacement along PC2 for each simulation is shown in (B).

In contrast to PC1 and PC3, the fluctuations of the CAP210 simulation along PC2 are much larger than those of the two B-clade simulations (Figure 3). In PC2, the motion of the bridging sheet is coupled to that of the outer domain spatially close to the α2-helix and to the motion of the loops in the outer domain leading to and returning from the bridging sheet. In addition, the outer domain exhibits moderate motion whereas the inner domain remains rigid. In other words, even though the dominant long-distance coupled motions of gp120 are conserved across different strains, there may be subtle strain-specific differences in these functional motions. The motion in PC2 might be more specific to certain gp120 sequences and could lead to sequence- or clade-specific antibody neutralization strategies. While we and others have established that the α2 helix of clade C gp120 is under higher immune pressure to evolve than the same region in clade B [8], [30], [32], [43], we illustrate here for the first time that sequence diversity in gp120 can also lead to subtle changes in the collective motions of this region in clade C gp120.

### Sequence dependent optimal path for communication in gp120

Both correlation and PCA of the gp120 simulations established that the long-distance coupled motions in gp120 are conserved to a large extent, and these motions dominate the intrinsic dynamics of gp120. Interestingly, the conserved motions in PC1 and PC3 couple the motions in the functional regions of gp120 – i.e., the CD4-receptor and the coreceptor binding sites. In addition, the PCA of these motions show that there are strain-dependent subtle changes to these long-distance coupled motions. It is often assumed that the global coupled motions in the protein occur due to transfer of energy between local contacts. Allosteric signatures involved in immune escape may also utilize these communication pathways. An important question is whether certain conserved aspects of these communication pathways can be targeted in immunogen design.

We utilized a dynamical network analysis method [63] to identify the routes associated with this signal transmission within gp120. This dynamical network analysis assumes that local coupled motion leads to long-distance coupled motion in the protein. In these networks, a node represents a residue in the protein, while edges connect nodes that are in contact for a majority of the simulation. The correlation of motion between residues in contact is used as a measure for the information transferred between these residues. The larger the edge distance, the lower the communication between the two nodes, since the nodes move more independently of one another during the simulation. In the dynamical network, the optimal pathway for communication is the one that is most coupled between distant sites in a protein. Suboptimal pathways refer to slightly less correlated pathways in the network that connect two regions in a protein. We initially analyzed whether the communication pathways in gp120 are conserved across all three strains to assess whether vaccine design strategies could be developed to reduce communication across these pathways.

Typically, the choices for the beginning and ending points for capturing a communication pathway are ligand binding sites or regions that are regulated in an allosteric fashion. In gp120, we chose one residue (HXB2# 353) in the CD4 binding loop as the beginning position (or source of information flow) due to its critical contribution to the CD4 receptor-binding site. In addition, the CD4-binding loop undergoes a conformational change upon binding to the CD4 receptor. We chose a second residue (HXB2 # 369) located at the C-terminal end of α2 helix as the endpoint, because this residue in the outer domain is the most distant from the CD4 binding loop (in terms of network distance). Also, conformations of outer domain varied less than the inner domain in all three simulations. The network in gp120 was examined to find the optimal (most coupled) and suboptimal paths for communication between the C-terminus of the α2 helix and the CD4-binding loop. In addition, sequence analysis of the α2 helix indicates that clade B and C sequences employ clade-specific mechanisms for immune escape and viral replication [8], [30], [32], [43]. Hence, we measured the most coupled pathway for communication between these regions in the dynamic networks generated for the three different sequences of gp120.

**Figure 4:**
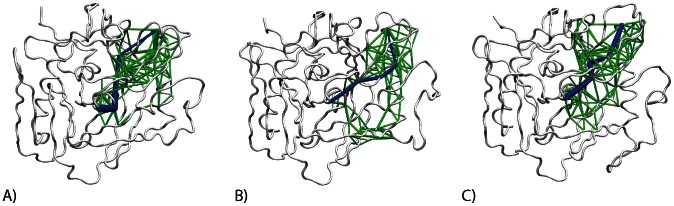
The optimal (blue) and suboptimal paths (green) for communication from the CD4 binding loop to the C-terminus end of the α2 helix for the (A) YU2 (B) HXB2 and (C) CAP210 networks.

A large number of suboptimal or pre-existing paths exist for communication between these two sites [80]. It was hypothesized in a study by Sethi *et al.* [63] that a sequence change could convert a suboptimal path to an optimal path in the mutated protein network. In other words, the optimal pathway for communication between two distant sites in the protein can display sensitivity to the protein sequence, and may be subject to selective pressure. We show here that the optimal pathway for communication between three different gp120 sequences does indeed vary (Figure 4 and Table 1). In other words, different sequences of gp120 utilize distinct pathways for communication between the α2 helix and the CD4 binding site. For example, in the case of YU2 communication passes through β9, β10, and β11 whereas it does not pass through these structural elements in HXB2 and CAP210. A number of studies have focused on defining a single optimal pathway for communication between distant sites without also considering the suboptimal pathways [81], [82]. However, we show here that a suboptimal pathway for communication in one sequence can serve as an optimal pathway for communication in another sequence of a homologous protein (Table 2).

**Table 1:** The residues along the optimal path for communication in the three networks.

YU2	HXB2	CAP210
F319 (353)	F353	F349 (353)
I251 (284)	E466	F471 (468)
L411 (453)	I360	L454 (454)
Q225 (258)	K362	P473 (470)
T339 (373)	S364	P359 (364)
P335 (369)	V372	T367 (372)
	P369	L364 (369)

The residues in parentheses show the corresponding HXB2 sequence numbering.

**Table 2:** The path distance of the shortest path in a particular network is shown for all three networks.

	Shortest Path in Network		
	YU2	HXB2	CAP210
YU2	2.57	3.18 (1235)	2.81 (46)
HXB2	3.95 (1217)	3.07	3.83 (288)
CAP210	3.38 (6675)	2.37 (1503)	1.92

The numbers in parentheses denote the rank number (in terms of path distance) of a path in that particular network. The rank of the optimal pathway in a particular network (diagonal terms in the table) is always 1 because it is the lowest path distance connecting the two nodes.

### Modularity of communication network in different sequences of gp120

**Figure 5:**
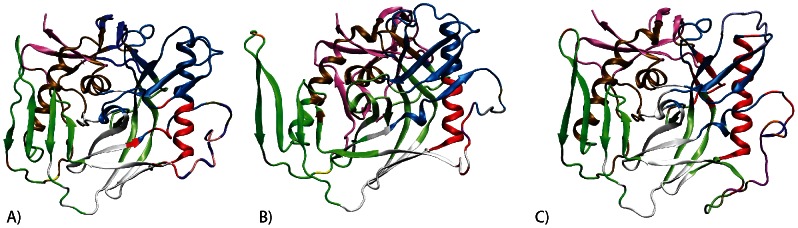
The modules or communities in the network for (A) YU2, (B) HXB2, and (C) CAP210 networks. The color represents the community that the residue belongs to (bridging sheet – green, αβ-bundle – brown, β-sandwich – magenta, parts of 6-stranded barrel – blue, V4 and α2 helix – red, 6- stranded barrel – white, interface of the two barrels in outer domain – lime).

The community analysis was carried out on networks built from all three MD simulations of gp120. The modules (communities) are very similar in all three networks (Figures 5, S6 and Table 3). There are seven major communities in each network. The bridging sheet forms one community (Figure 5, green), while the α1 helix forms a second community along with the α5 helix (Figure 5, brown), which is close to the interface with the outer domain. In addition, the five-stranded β-sandwich forms the third major module in the inner domain (Figure 5, magenta). Due to the β1 strand unfolding during the timescale of the simulation, there is some splitting of the β-sandwich into two communities in the YU2 simulation, but to a large extent, the communities are conserved in this region between the different simulations. The outer domain is split into four major communities. In YU2 and HXB2, one community is formed by the C-terminal half of the α2 helix and the six-stranded barrel that interacts with it (Figure 5, blue), while a second community is formed by parts of the V4 loop and the N-terminus half of the α2 helix (Figure 5, red). Parts of the six-stranded barrel form the third community (Figure 5, white), and an additional community is formed by the rest of the seven-stranded barrel (Figure 5, lime). This community structure is highly conserved across all three sequences of gp120 that we have simulated.

**Table 3:** Comparison of the YU2 and CAP210 networks to the HXB2 network based on the subdomains in the protein.

Community	YU2				CAP210			
	AdjMat	Conn	clCoeff	maxAdjRat	AdjMat	Conn	clCoeff	maxAdjRat
β-sandwich	0.33	0.42	0.21	0.54	0.86	0.90	0.89	0.63
αβ-bundle	0.52	0.61	0.25	0.22	0.70	0.77	0.48	0.42
Bridging sheet	0.22	0.16	−0.04	−0.07	0.42	0.34	0.11	0.08
6-stranded barrel & α2 helix	0.86	0.88	0.70	0.68	0.69	0.62	0.36	0.34
7-stranded barrel	0.72	0.82	0.60	0.58	0.66	0.68	0.33	0.32

Each column measures the correlation in different properties of the dynamical network based on the simulation. The correlations in adjacency matrix (AdjMat), connectivity (Conn), and maximum adjacency ratio (maxAdjRat) measure similarities in pairwise connections of each node in the subdomain, while the correlation in clustering coefficient (clCoeff) is a measure of clustering of homologous residues in different sequences of gp120.

Interestingly, in the CAP210 network, the six-stranded barrel forms a community (blue), but the α2 helix is not a part of this community. Instead, the α2 helix forms a separate community of its own (red). In other words, there are subtle changes in the CAP210 modules within this conserved structure. We also observed differences in the network in this region when we compared the modules (Table 3). This is consistent with the PCA from the three simulations discussed above and with the concept that B-clade (such as YU2 and HXB2) and C-clade (such as CAP210) envelopes use different mechanisms for immune escape near the C-terminus of the α2 helix [30], [32].

### Preservation and disruption of communication modules within gp120

Conservation of subsections of the network is quantified based on the correlation in network properties (see Methods and [83]) within each subdomain in the protein (Table 3). A correlation value of +1 in the intramodular property across two different networks implies perfect conservation of the module in both networks, while a value of 0 denotes no conservation in the modules. As described above, the subdomains in the inner domain undergo changes during the simulation. Due to this relative instability of inner domain of unliganded gp120 in the CD4-bound conformation, the modules (especially, the bridging sheet) show more variability in network properties across the three different proteins. In addition, the YU2 simulation undergoes further changes during the timescales of our simulations; the β1 strand breaks away from the β-sandwich and the β2 and β3 strands break away from the bridging sheet. As a result, it is difficult to distinguish these inner domain modules with regard to the phylogenetic groups, as it appears that the CAP210 network is similar to the HXB2 network. In depth analysis of such distinctions between networks can be useful in deducing differences in macrophage and non-macrophage tropic Env proteins [66].

The communities are highly conserved in all three gp120 core sequences studied here, and these modules are more conserved in the outer domain of gp120 than the inner domain. Furthermore, the network in the outer domain is more highly conserved between the YU2 and the HXB2 networks as compared to the CAP210 network. This is consistent with the differential splitting of the α2 helix in this region into different communities in YU2 and HXB2 but not in CAP210 (Figure 5). The differences between the CAP210 and the HXB2 networks in these modules are more pronounced in correlations of clustering coefficient and maximum adjacency ratio (Table 3), as these are more sensitive to the overall structure of the network than connectivity and adjacency matrix. Further analysis suggests that the network in the α2 helix is more similar between the two B-clade sequences simulated here than either of the B-clade networks are to the C-clade network.

### Identification of residues that form inter-modular edges – “hotspots”

Nodes in the same community are highly interconnected and can communicate with one another very efficiently through multiple routes. Nodes belonging to different communities have fewer connections between them and could form a conduit for information transfer in the network. A previous study by Sethi et. al. found that some residues occurred in most of the suboptimal paths connecting distant regions and were highly conserved through evolution [63]. If communication through the inter-modular contacts is reduced or eliminated, the network becomes fragmented and the modules become independent of one another [63], [80]. Therefore, we term residues that form these inter-modular edges ‘hotspots’ (listed in Table S2).

Initially, we considered whether these hotspots occurred in regions where broadly neutralizing antibodies bind. There are two sites that are targeted by neutralizing antibodies in the core of gp120 – the CD4 binding site and the bridging sheet. Antibodies that bind to the CD4 binding site compete with binding to the CD4 receptor, thus blocking viral entry into host cells. Thus, antibodies that target this conserved and functionally critical region of gp120 are both broadly neutralizing and highly potent [1], [84]–[89] and the gp120 residues required for CD4 binding and recognition by CD4 binding site antibodies are well defined and often overlapping [1], [85], [90]. However, variation in sequences distant from the CD4 binding site can also influence sensitivity to neutralization at this site, but the exact mechanisms by which these mutations lead to immune escape are unknown [20], [28]–[32]. Antibodies that bind to the highly conserved bridging sheet can block viral entry into host cells by competing with the coreceptor for binding to gp120; however, neutralization by antibodies that target the bridging sheet is limited because this structure is only exposed after gp120 binding to the CD4 receptor [91]. It is likely that these antibodies are present in infected individuals and that they impose strong selective pressure on HIV-1 to remain dependent on CD4 for entry [92].

To investigate hotspots in terms of the CD4 and coreceptor binding sites, we based our analysis on four high-resolution crystal structures of monoclonal antibodies (b12, b13, F105, and VRC01) bound to the CD4 binding site [1], [85], [90] and one structure of monoclonal antibody 17b bound to the bridging sheet in gp120 [92]. We searched for the hotspot residues identified using our approach within the antibody-binding interfaces of gp120 in these structures. We noticed that multiple surface exposed hotspots occur close to the CD4 binding loop and the bridging sheet regions. In each of the five antibody-bound structures, we found between 4 and 8 hotspot residues at the interface of each antibody with gp120 (Table S3). In other words, several hotspot residues from each network are targeted during antibody binding to gp120. Thus, targeting residues that are critical to the integrity of the gp120 network may contribute to the high potency and breadth of these antibodies.

Next, we considered whether these hotspots occur in neutralization signature sites or regions under high selective pressure. A number of sequence-based studies have been performed recently by our group and others to identify genetic signatures associated with immune escape of HIV-1 in a population of infected individuals and to map out the effect of mutations on neutralization sensitivity to monoclonal antibodies [20], [28]–[32]. In particular, we performed a combination of experimental and bioinformatic analyses identified the genetic signatures associated with escape from the monoclonal antibody b12 that binds to the CD4 binding site [20]. In addition to signatures located at the interface of gp120 and b12, genetic signatures that were distant to the interface were also associated with escape from b12 neutralization. Of these distant signatures, only one residue (E268) was located within the gp120 core (i.e. not in a hyper-variable domain) was associated with immune escape from b12 antibody. Furthermore, residue E268 is located approximately 30 angstroms from the b12-gp120 interface. The fact that we also identified E268 as a critical residue on the CAP210 network here argues that a mutation at this position could impact the flow of information within gp120, in addition to decreasing b12 binding. Another study identified residues 456, 458, and 459 as neutralization signatures against the NIH45-46 antibody that also targets the CD4-binding site [93]. These residues occur distant from the antibody-binding site and residues 457 and 459 were also identified as hotspots in the CAP210 network in this study.

As immune escape mechanisms can be context (or clade) specific, studies have focused on identifying regions under high positive selection in a population infected with either the clade B or C virus [31], [48], [94], [95]. We recently performed a comparison of the sequence and structural characteristics of different regions of gp120 in clades B and C, and found that the V4 loop and α2-helix exhibit key clade-specific patterns in variation with antigenic implications [30]. A recent study with clade C viruses also reported that three residues (393, 397 and 413) in the V4 loop were associated with greater neutralization sensitivity [29], and two of these residues are hotspots in the CAP210 (clade C) network. We also reported evidence that five residues within the α2 helix were under high immune pressure to evolve rapidly in the clade C (335, 336, 337, 343, and 350) [30]–[32]. The residues 334, 335, and 349 in α2-helix were also identified as hotspots for communication in the clade C CAP210 network. In clade B, only residues 333 and 335 were identified for HXB2 in the N-terminus of α2-helix and residues 338 and 342 were identified for YU2 sequence.

The genetic signatures identified in the above studies do not correspond to commonly known neutralizing antibody binding sites in gp120, are distantly located to the broadly neutralizing antibody binding sites, and are modulated in an allosteric manner. Interestingly, some of these signatures affect antibody binding to gp120 in the CD4-binding site and/or the coreceptor-binding site presumably without affecting the entry function of gp120. While the exact mechanism(s) utilized by these residues to modulate antibody binding at distant sites remains unknown, we propose that these signature residues could mediate antibody escape by an allosteric mechanism via the gp120 communication network. In other words, if the virus can mediate antibody escape at a highly conserved, functional domain by making a change in a region that is more tolerant to diversity, then Env function is much less likely to be disrupted. Defining the biological contributions of these hotspots will require additional studies, some of which will need to include the full-length, glycosylated gp120-gp41 trimer. Nevertheless, the alteration of communication pathways in an Env immunogen could cause subtle changes in gp120 conformation that may in turn alter epitope exposure. The CD4 binding site in particular may be amenable to such interventions.

### Hotspots in regions of binding leverage – an independent validation

Finally, we independently verified that the inter-modular edge hotspot residues occur close to regions that affect the long-distance coupled motion of gp120 by using an alternate methodology based on binding leverage calculations. The binding leverage of a ligand-binding site measures the additional amount of stress introduced into the ligand-bound protein due to coupled motion along the ten lowest energy (or most dominant) normal modes of the *apo* protein [27], [96]. The binding of an antibody to a particular site on gp120 introduces new ligand-mediated contacts between residues in this region of the protein. The binding leverage measures the coupling between ligand binding and the functional dynamics of the protein. While mutations in the sequence of gp120 can lead to an increase or reduction in the number of contacts near the mutation site, binding leverage only measures the perturbations due to the addition of contacts when a ligand is introduced to the protein. The normal modes are calculated using an elastic network model and the collective motions in the lowest energy normal modes often dominate the motions involved in the regulatory conformational changes of the protein [27], [96]. Regions with high binding leverage could potentially have a greater effect on the motions in these normal modes after a perturbation (induced in the form of a ligand, or more generally, by a change in local structure or sequence) is introduced in this region. This approach has been utilized to accurately predict the allosteric sites in a known set of proteins [27], [96].

Even though the normal modes and binding regions are calculated based on the position of Cα atoms in gp120, a high correspondence between hotspots identified using the network analysis and regions of high binding leverage was observed. Between 70–80% of the regions identified during the binding leverage simulations contain at least one hotspot residue. In all three sequences, multiple hotspot residues tend to occur in the core of ligand binding sites with high-to-moderate binding leverage as compared to sites with low binding leverage (Tables S4, S5, S6 and Figure S7). Hotspot residues located in the CD4 binding site, the bridging sheet, and the β-barrel in the inner domain are conserved across all three structures and also have moderate to high binding leverage. In addition, a number of regions near the interface of the inner and outer domains contain conserved hotspots across all three networks and correspond to regions with moderate binding leverage. With the exception of one residue, the hotspots in all three networks have finite binding leverage and correspond to regions that could potentially affect the collective motions in the lowest normal modes of gp120.

## Discussion

Allosteric signal transmission involves the transfer of energy, leading to the communication of dynamic information between distant regions of the protein. This energy flows anisotropically through the residues in the protein leading to coupled motions in distant regions of the protein [97]. The HIV-1 Env gp120 protein employs an allosteric mechanism that is essential for entry of the virion into a CD4+ T-cell as well as for immune evasion. Here, we utilized a network analysis method based on the local correlation of motion between contacts in the protein [63] to identify the routes associated with this signal transmission within gp120. This dynamical network assumes that local coupled motion leads to global allosteric changes in gp120. We combined this network analysis approach with molecular dynamics simulations to deduce the conserved and variable features of the communication pathways in three known HIV-1 envelope gp120 protein from two different clades, B and C. The present study is also the first to investigate whether (i) the modules in a protein with the same structure change with sequence differences and (ii) changing the community structure in a protein can lead to different allosteric pathways.

First, we established the existence of long-distance coupled motions in gp120 with correlation and principal component analyses. These analyses demonstrated that the coupled motion between distant functional sites on gp120 is conserved and dominates its motions. Furthermore, these motions are reminiscent of allosteric regulation. We then utilized a network theory based approach to study the conserved and variable features of communication in three different gp120 networks, representing two HIV-1 subtypes. We show that many different pathways exist for communication between spatially distant sites in gp120 and a suboptimal pathway in one strain can serve as the optimal pathway in another strain (Tables 1 and 2). While the long distance coupled motions are highly conserved across the three gp120 cores considered, the shortest route for communication between spatially distant sites in gp120 varied with the sequence. Our analysis indicates that HIV-1 gp120 could retain its function and escape from antibody neutralization through mutations that allow it to utilize one of the suboptimal paths if the shortest pathway becomes blocked. This finding is consistent with the observation that genetically distinct, co-circulating HIV-1 variants within an individual commonly use different escape pathways to resist neutralization by the contemporaneous autologous antibody pool [6], [98]. More often than not, these escape pathways appear to protect conformational epitopes. Hence, blocking or altering dominant and suboptimal pathways for communication in gp120 should also be considered in vaccination strategies to increase exposure and/or immunogenicity of conserved epitopes to increase neutralization breadth.

Due to the redundancy of communication pathways in gp120, a natural question that arises is whether any conserved aspects of the network can be utilized for immunogen design. Here we investigated the modules of the network to answer this question. Residues within a module (also called communities) are highly connected, but residues in different modules contain relatively fewer edges between them. Thus, each community in the dynamical network is made up of residues that are in contact with each other and move in a correlated fashion during a MD simulation [63]. In contrast, the inter-modular junctions form conduits for information flow in the network, and by reducing information flow through these edges, one could potentially impose a larger impediment to communication through a protein network. An important question that we address here is whether the modules in dynamical networks are conserved across viral evolution and can therefore be targeted for therapeutic intervention. The community analysis of the network from our study revealed that modules were conserved across the three different gp120 strains. However, there were subtle changes in these communities, for example in the α2 helix region, that could lead to different allosteric immune evasion mechanisms or immunogenic properties in envelopes from phylogenetically distinct groups or clades. This is consistent with our previous studies demonstrating distinct mutational patterns in and around α2 between clade B and C gp120 [30]. These findings also support that vaccines designed for certain populations should include strategies that consider the dominant circulating clade or recombinant form.

Given the highly conserved modular nature of the gp120 network, the interface of these communities pointed to the presence of hotspots for long-distance communication in the protein. These hotspots exist at the junctions between modules in the network, and the communication between residues in two different modules has to flow through relatively fewer inter-modular edges. In this study, we found that a number of surface-exposed hotspots occur close to the functionally important CD4 binding loop and the bridging sheet region. This could be one of the reasons why some antibodies that target the CD4 binding site region exhibit broadly neutralizing character. Importantly, we show that these hotspots occur at residues that are part of well-defined epitope in gp120, as well as in sites distal to these epitopes that have been associated with neutralization resistance or immune escape. Furthermore, a number of hotspots occur along the α2 helix, and these residues were found previously to be under high selective pressure in a clade-specific manner in our earlier studies [8], [30], [32], [43]. Importantly, we verified the occurrence of these hotspots using an independent approach. This second analysis demonstrated that the perturbations near hotspots could potentially influence long-distance coupled motions (lowest energy normal modes) that dominate the intrinsic dynamics of gp120 core. Even though the dynamical network method as done in this study is more computationally intensive than the binding leverage-based method, the former is better suited to study sequence-specific allosteric communication mechanism compared to the Cα-atom based latter approach. Our studies suggest that even in the presence of multiple pathways for communication between distant regions in gp120, the conduits for information flow (hotspots) that we have defined could be exploited in new immunogen design strategies.

Finally, these communication hotspots could potentially be exploited to interfere with the flow of information across the allosteric network in gp120. The HIV-1 envelope has evolved multiple mechanisms to maintain an inherent level of neutralization resistance by protecting its most vulnerable and well conserved targets. The novel and rational immunogen design approach that we introduce here could be used in envelope-based vaccine strategies to focus the immune response on critical hotspot residues, or mutate those residues directly to expose conserved epitopes in gp120 (i.e. the CD4 binding site), in an effort to induce antibodies with neutralization breadth. Furthermore, this approach could inhibit long-distance immune escape pathways within gp120 should breakthrough infection occur by inducing antibodies against regions that contain these hotpots. Here, one can target vaccine-induced antibodies to hotspots to minimize the potential for immune escape via long-distance allosteric communication following a breakthrough infection. Many of the hotspots lie along the suboptimal paths that connect distant regions in gp120. If information flow through these hotspots could either be reduced or removed completely perhaps by mutating them, the allosteric network would become fragmented and the modules would function independently of one another. This could also lead to changes in conformation that expose otherwise hidden epitopes and increase neutralization breadth. Thus, this network-based approach could reduce the capacity of the HIV-1 envelope to shield its vulnerable neutralization targets, producing more effective immunogens.

## Methods

### Homology modeling

The missing regions of the structures for the YU2, HXB2, and CAP210 sequences (PDB accession numbers 1G9M [71], 1RZK [70], and 3LQA [44]) lacking the V1–V3 loops were modeled using the MODELLER program [99]. The core of gp120 was similar in all these structures. It should be noted that 3LQA is the only high-resolution structure of a clade C gp120 sequence, while multiple clade B gp120 structures exist. Multiple templates were used because it has been shown that this creates a high-quality homology model. During modeling, disulfide constraints were added for the conserved cysteines present in all gp120 sequences. All sequence alignments used for modeling templates were based on sequences in the HIV-1 database (www.hiv.lanl.gov).

### Molecular dynamics simulations

The starting conformations for the long timescale all-atom MD simulations were modeled using MODELLER [99] as described above. The protein was solvated in TIP3P water molecules [100] and neutralized in 150 mM NaCl salt. The MD simulations of the solvated proteins were performed using NAMD2 [101] with the CHARMM27 force field [102]. The protein was initially minimized and then heated to 298K with constraints added during these steps similar to the protocol in [103]. All simulations were performed with periodic boundary conditions using the NPT ensemble with pressure set to 1 atmosphere and temperature set to 298K. The pressure and temperature were maintained using the Langevin piston and the Langevin theromostat respectively. Electrostatics were calculated with the particle mesh Ewald method [104]. The van der Waals interactions were calculated using a switching distance of 10 Angstroms and a cutoff of 12 Angstroms. All the production runs were performed with 2 fs time step using the RATTLE [105] and SETTLE [106] algorithms. The proteins were equilibrated for 10 ns, and the initial burst in RMSD converged within this period. The YU2, HXB2, and CAP210 simulations were performed for a further 600 ns each. The number of atoms in each system was approximately 50,000 atoms. The coordinates were saved once every 1 ps in each simulation.

### Correlation

Correlations between all of the residues in gp120 were analyzed for the 600 ns production run using the normalized covariance:
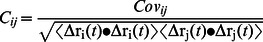
where 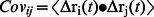 denotes the covariance in motion of the Cα-atoms of residue *i* and *j*; while 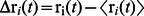. The correlation matrix is also called the dynamic cross correlation matrix. The value of *C_ij_* is between the values of −1 and 1. If *C_ij_* = 1, then the residues are moving in a correlated fashion (same direction) during the simulation, while *C_ij_* = −1 implies that the residues are moving in an anticorrelation fashion (or in opposite directions). Residues that move independently of one another have a correlation value close to zero. However if residues move in a correlated fashion in perpendicular directions, their correlation value will also be close to zero. The frames are saved at an interval of every 1 ps, and a total of 600,000 frames were analyzed for the correlation matrices of each simulation.

### Principal component analysis

To investigate the collective behavior within the complex, a standard principal component analysis (PCA) of the motions of the C**α** atoms during the equilibration was performed as implemented in the program CARMA [107]. The unnormalized covariance matrix, **Cov** defined above, was diagonalized during PCA. The largest eigenvalues and their accompanying eigenvectors, capture the largest fraction of the observed variance in the motion. The contribution of each eigenvector to the observed motion is obtained using the projection matrix. On projecting the data from principal component *i* onto the Cartesian coordinates, the RMSD of each residue was calculated due to the *ith* principal component. The RMSD per residue plots give an estimate of regions that are highly coupled due to the *ith* principal component.

### Network analysis

A network is defined as a set of nodes with connecting edges. Each amino acid residue in the protein is represented by a node in the network. Edges connect pairs of nodes if the corresponding residues are in contact, and 2 nonconsecutive monomers are said to be in contact if any heavy atoms (non-hydrogen) from the 2 monomers are within 6.5 Å of each other for at least 75% of the frames analyzed. The edges are weighted by the correlation in motion between the residues: 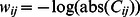 [63]. The (anti-) correlation in motion is used as a measure for information transfer between the two residues in contact.

### Shortest paths and suboptimal paths

The length of a path *D_ij_* between distant nodes *i* and *j* is the sum of the edge weights between the consecutive nodes (*k,l*) along the path: 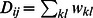. The shortest distance *D_ij_* between all pairs of nodes in the network is found by using the Floyd–Warshall algorithm. The betweenness of an edge is the number of shortest paths that cross that edge.

Although the shortest path is the most dominant mode of communication between the nodes, the number of paths within a certain limit of the shortest distance is a measure of the path degeneracy in the network. All suboptimal paths for communication between the active site and the identity elements are determined in addition to the shortest path. The tolerance value used for any alternate path to be included in the suboptimal path was 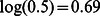, which is close to the average protein edge weight.

### Community analysis

The network contains modules or communities of nodes that are more densely interconnected to each other than to other nodes in the network. The community structure is identified by using the Girvan–Newman algorithm [108]. In this algorithm, the shortest paths between all pairs of nodes in the network are calculated. The betweenness of an edge in the network is defined as the number of shortest paths that pass through it. The Girvan-Newman algorithm uses a top down approach to iteratively remove the edge with the highest betweenness and recalculate the betweenness of all remaining edges until none of the edges remain. The optimum community structure is found by maximizing the modularity value Q, which is a measure of difference in probability of intra- and intercommunity edges. As the algorithm divides the network into increasingly smaller communities, the modularity score is measured for each community division, and the maximum value corresponds to the optimal community distribution of the network. More recently, a number of algorithms have been developed that explore different strategies for dividing a network into community structures, but they are more complex and provide only subtle differences in the community architecture of these proteins. The variation of cutoffs used to define contacts was investigated by Sethi, et al., 2009 and showed that changes in the parameters (75% of frames and 4.5 Angstroms cutoff between any pair of heavy atoms in residues) defining the network contacts led to minor changes in the community distribution of the network.

To compare the sensitivity of different subdomains in the networks to the sequence of the protein, we calculated module preservation statistics defined in [83]. Briefly, the module preservation statistics measure the preservation of connectivity and weights of these connectivities between nodes within the modules in different networks. These calculations were performed only over the core of the protein (any position that was gapped in any of the structures were not considered to be part of the core), as the number of nodes for each subdomain has to be constant in the different networks. The HXB2 network was considered as the reference network for this analysis. However, the trends in Table 3 are independent of the reference network. There are four network measures that are considered in this analysis:

1. Intramodular adjacency matrix: The adjacency matrix is a square matrix (elements denoted as 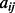) that encodes the network connection between nodes *i* and *j*. When 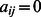, the nodes are not connected. If the number of nodes in the network is equal to *n*, then the number of nondiagonal elements in the adjacency matrix is 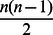. The intramodular adjacency matrix is the adjacency matrix of all nodes within a given module. The correlation in intramodular adjacency matrix (AdjMat in Table 3) between two networks is a measure of the similarity of the strength of connections within a module between both networks. The connections between modules are neglected in this method.
2. Connectivity: The *connectivity* (also known as degree) *k_i_* of node *i* is defined as
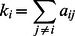
The connectivity of node *i* measures its connection strength with other nodes. The intramodular connectivity measures its connection strength to other nodes within the same module. The correlation in intramodular connectivity (denoted as Conn) in Table 3 measures the similarity in connection strengths of each node within a module across both networks.
3. Maximum Adjacency Ratio (MAR): The Maximum Adjacency Ratio (MAR) *MAR_i_* of node is defined as:
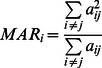
The maximum adjacency ratio is helpful in analyzing connectivity patterns in weighted networks. The correlation in MAR (denoted as MAR in Table 3) measures the similarity in connectivity patterns of each node within a module across both networks.
4. Clustering Coefficient: The clustering coefficient of node *i* is defined as
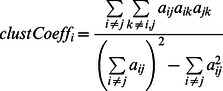
The clustering coefficient of node *i* is a measure of the probability of finding two nodes *j* and *k* connected when both of them are connected to node *i*. The correlation in intramodular clustering coefficient was calculated to measure the similarity in clustering of nodes in each module between different networks.

### Binding leverage

The calculation of binding leverage initially involves the identification of potential ligand binding sites in the protein as detailed in [27]. The ligand binding sites were identified using Monte Carlo docking simulations to the protein represented by its Cα-atoms. The probe contained 6 atoms in these simulations and the bond angles in the probe were allowed to vary between 90 and 180 degrees. The probe and protein interacted via a square well potential which was attractive for Cα-Cα distances between 5.5 and 8 Å. Distances shorter than 4.5 Å were forbidden. The probe binding sites were identified in 1000 docking simulations each containing 10000 Monte Carlo steps. Binding leverage is defined as the amount of distortion in the probe location due to the motions in the lowest ten normal modes. The normal modes were calculated using the anisotropic network model made from the Cα atoms in the protein [42]. Springs were introduced between any two residues in contact within the protein (default contact distance cutoff of 15 Å). The probe introduced additional contacts in the protein near the probe binding site. A spring was placed between all residue pairs in a probe location whose interconnecting lines pass through the ligand. The binding leverage was calculated as the change in potential energy due to the distortion in these springs induced by the first ten normal modes in the protein.

## Supporting Information

Figure S1 The optimum fit for gp120 trimer with the SIV monomer conformation onto the cryo EM density map for native gp120 trimers [77]. We generated the fit using Molecular Dynamics Flexible Fitting [109] starting with the SIV conformation for gp120 and the cryo EM density. Similar results were shown in [77] based upon rigid docking of the SIV structure to the cryo EM map of the trimer. The core of gp120 is shown in blue, while V1, V2, and V3 loops are shown in green, red, and yellow respectively. White represents glycans added to the V1/V2 loop. The mismatch of the fit is shown by density in the middle that is not filled by the protein in this conformation.
(TIF)

Figure S2 The average overlap of covariance matrix for different intervals (8 ns to 300 ns) with the final covariance matrix calculated over the 600 ns trajectory.
(TIFF)

Figure S3 Flexibility of gp120 during the MD simulations of YU2 strain. (A) The protein is colored by the RMSF per residue. Blue represents residues that are rigid during the simulation while red represents residues that are flexible during the simulation. (B) Comparison of the RMSF per residue during three different MD simulations and one FIRST simulation.
(TIF)

Figure S4 RMSD of the conformations along the 600 ns trajectory in the YU2 simulation. (A) The plot is colored by the RMSD between any two frames during the simulation. Blue represents conformations with low RMSD (high structural similarity) and red represents conformations with high RMSD (low structural similarity). (B) The RMSD of each subdomain of the protein (as compared to the initial conformation) during the simulation is plotted as a function of time.
(TIF)

Figure S5 The RMSF per residue during the (A) HXB2 and (B) CAP210 during the 600 ns simulation.
(TIF)

Figure S6 The protein colored by the community each residue belongs to. The backbone of the protein is shown in tube. Again, it would be good to label some of the structures.
(TIF)

Figure S7 The average number of hotspot residues in 10 blocks of binding sites is plotted. To reduce the noise, the binding sites were arranged by decreasing binding leverage and were divided into 10 blocks. Block 1 contains the sites with the highest binding leverage (10%) while block 10 contains 10% of the sites with the lowest binding leverage. Higher numbers of hotspot residues occur in the sites with highest binding leverage in Hxb2 and YU2 simulations while a higher number of hotspots occur in sites with moderate binding leverage in the CAP210 simulation. In all three simulations, the sites with the lowest binding leverage tend to have a smaller number of hotspot residues.
(TIF)

Table S1 The overlap of the covariance matrix between different simulations. The values of overlap are between 0.5 and 1.0, indicating that the major coupled motion in the protein is similar for the three different gp120 sequences.
(DOCX)

Table S2 Hotspots in YU2, HXB2, and CAP210 networks.
(DOCX)

Table S3 The hot spot residues in each network that occur at the interface (within 4.5 Angstroms of antibody) of antibody binding sites are listed. The residue number shown in the table refers to the HXB2 sequence numbering while the residue name corresponds to the strain of gp120 in the PDB structure.
(DOCX)

Table S4 Binding Leverage of hot spot residues identified using community analysis from YU2 simulation. The binding leverage of a residue refers to the highest binding leverage of a site in which the hotspot residue is present.
(DOCX)

Table S5 Binding Leverage of hotspot residues identified using community analysis from CAP210 simulation. The binding leverage of a residue refers to the highest binding leverage of a site in which the hotspot residue is present.
(DOCX)

Table S6 Binding Leverage of hotspot residues identified using community analysis from HXB2 simulation. The binding leverage of a residue refers to the highest binding leverage of a site in which the hotspot residue is present.
(DOCX)

Text S1 Additional results and methods are presented in Text S1.
(DOCX)
