# Supplementary figures and images for: A Mechanistic Understanding of Allosteric Immune Escape Pathways in the HIV-1 Envelope Glycoprotein
Source: PLoS Comput Biol. 2013 May 16;9(5):e1003046. doi: 10.1371/journal.pcbi.1003046 (PMC3656115; doi:10.1371/journal.pcbi.1003046)

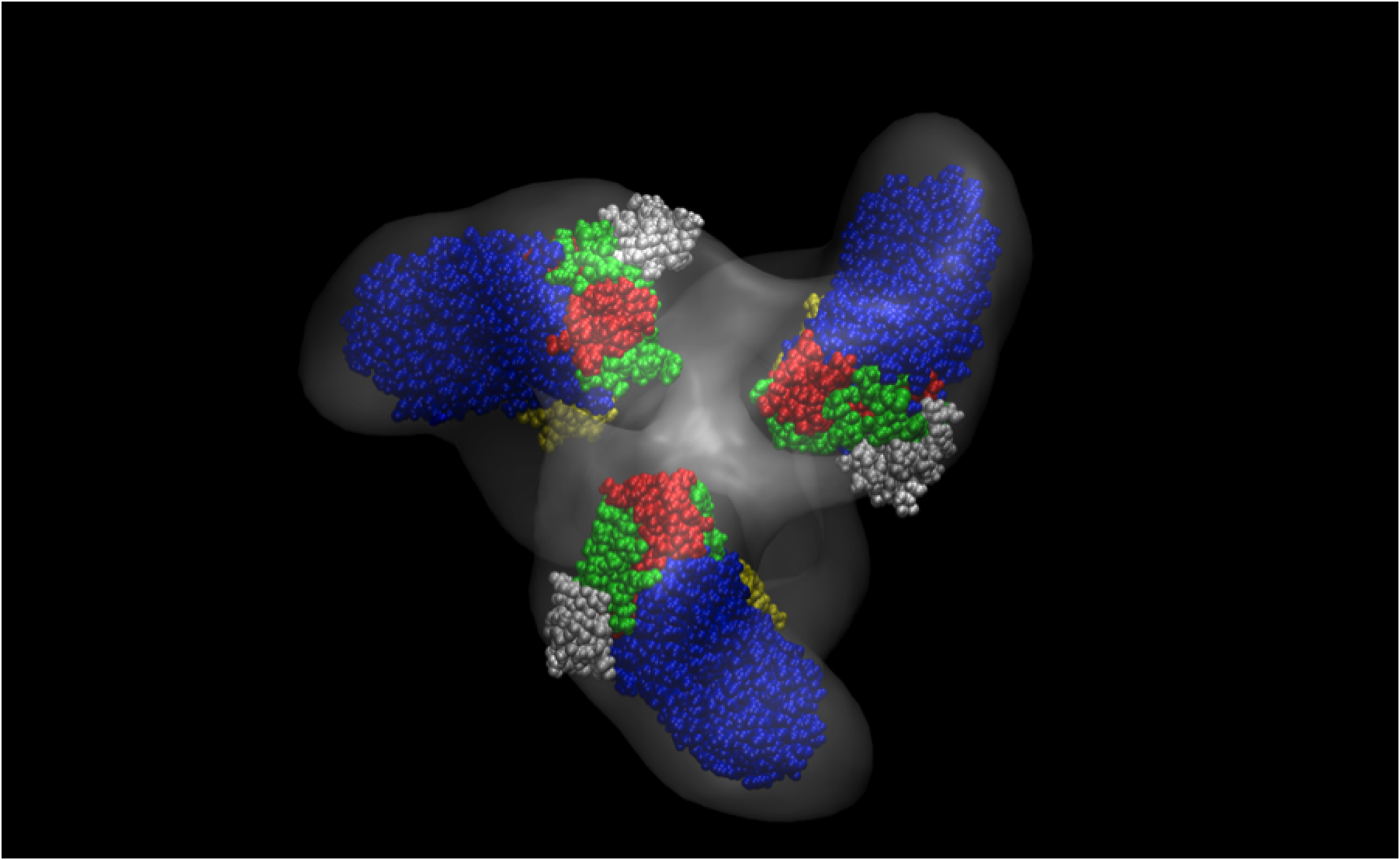

Supplement: Figure S1 — The optimum fit for gp120 trimer with the SIV monomer conformation onto the cryo EM density map for native gp120 trimers [77]. We generated the fit using Molecular Dynamics Flexible Fitting [109] starting with the SIV conformation for gp120 and the cryo EM density. Similar results were shown in [77] based upon rigid docking of the SIV structure to the cryo EM map of the trimer. The core of gp120 is shown in blue, while V1, V2, and V3 loops are shown in green, red, and yellow respectively. White represents glycans added to the V1/V2 loop. The mismatch of the fit is shown by density in the middle that is not filled by the protein in this conformation. (TIF) [file pcbi.1003046.s001.tif]

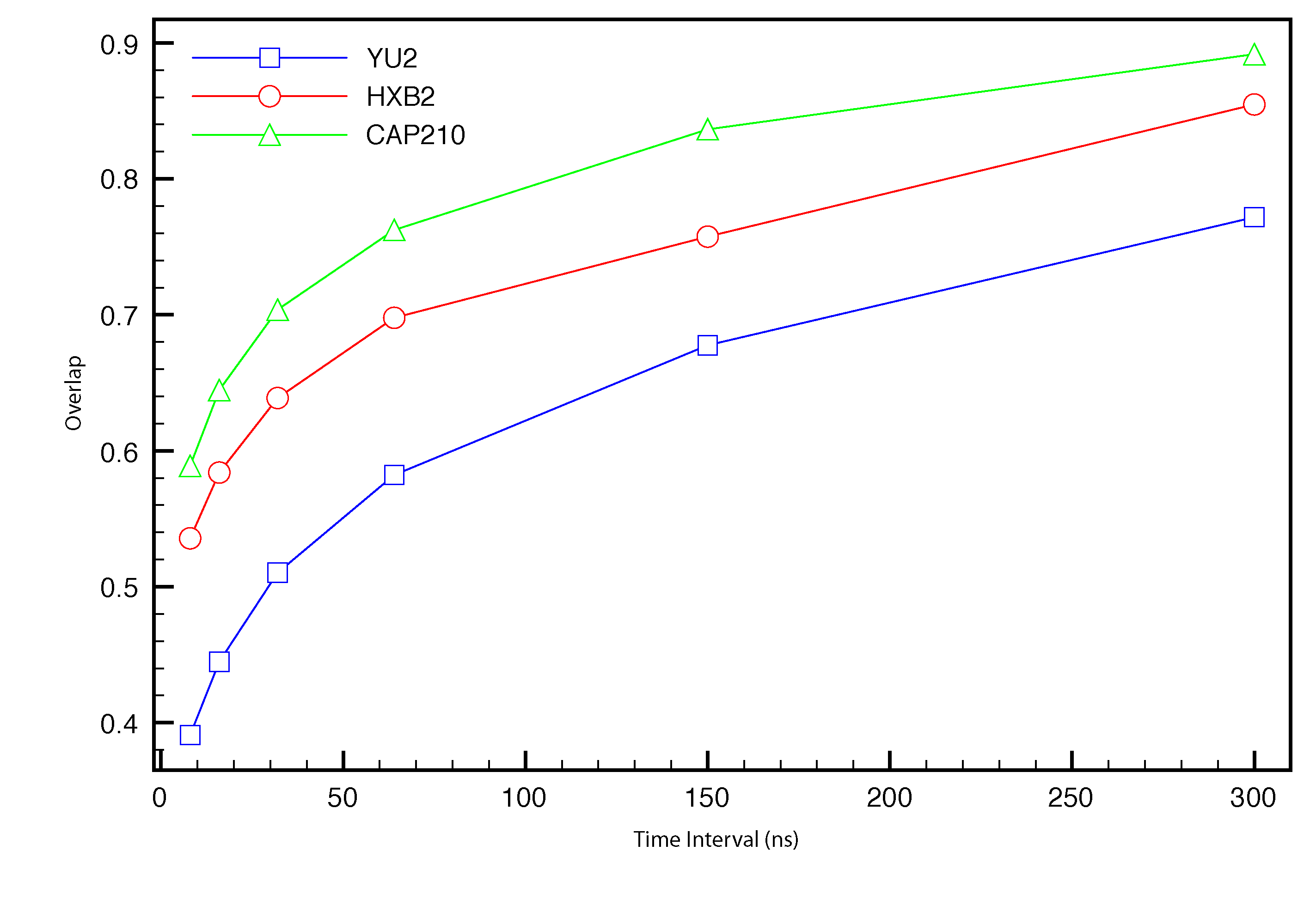

Supplement: Figure S2 — The average overlap of covariance matrix for different intervals (8 ns to 300 ns) with the final covariance matrix calculated over the 600 ns trajectory. (TIFF) [file pcbi.1003046.s002.tiff]

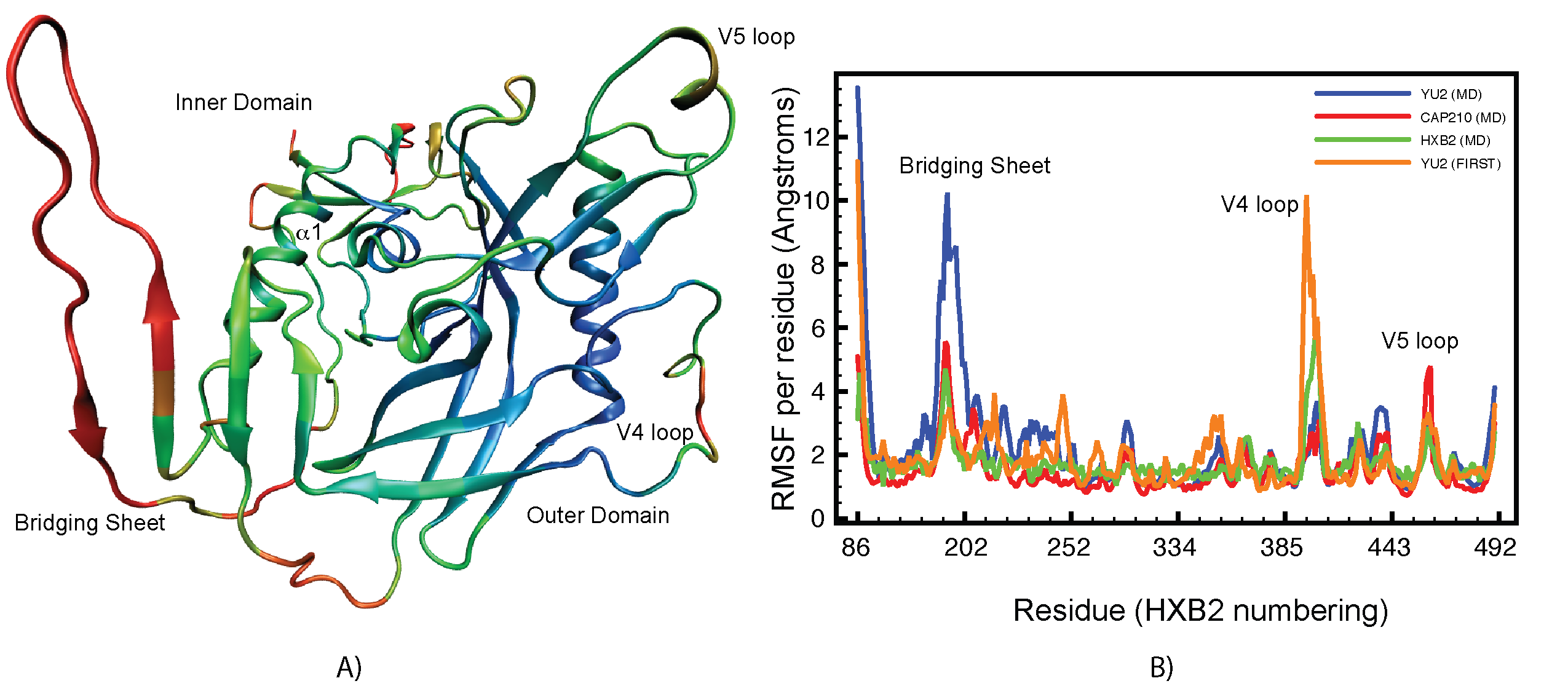

Supplement: Figure S3 — Flexibility of gp120 during the MD simulations of YU2 strain. (A) The protein is colored by the RMSF per residue. Blue represents residues that are rigid during the simulation while red represents residues that are flexible during the simulation. (B) Comparison of the RMSF per residue during three different MD simulations and one FIRST simulation. (TIF) [file pcbi.1003046.s003.tif]

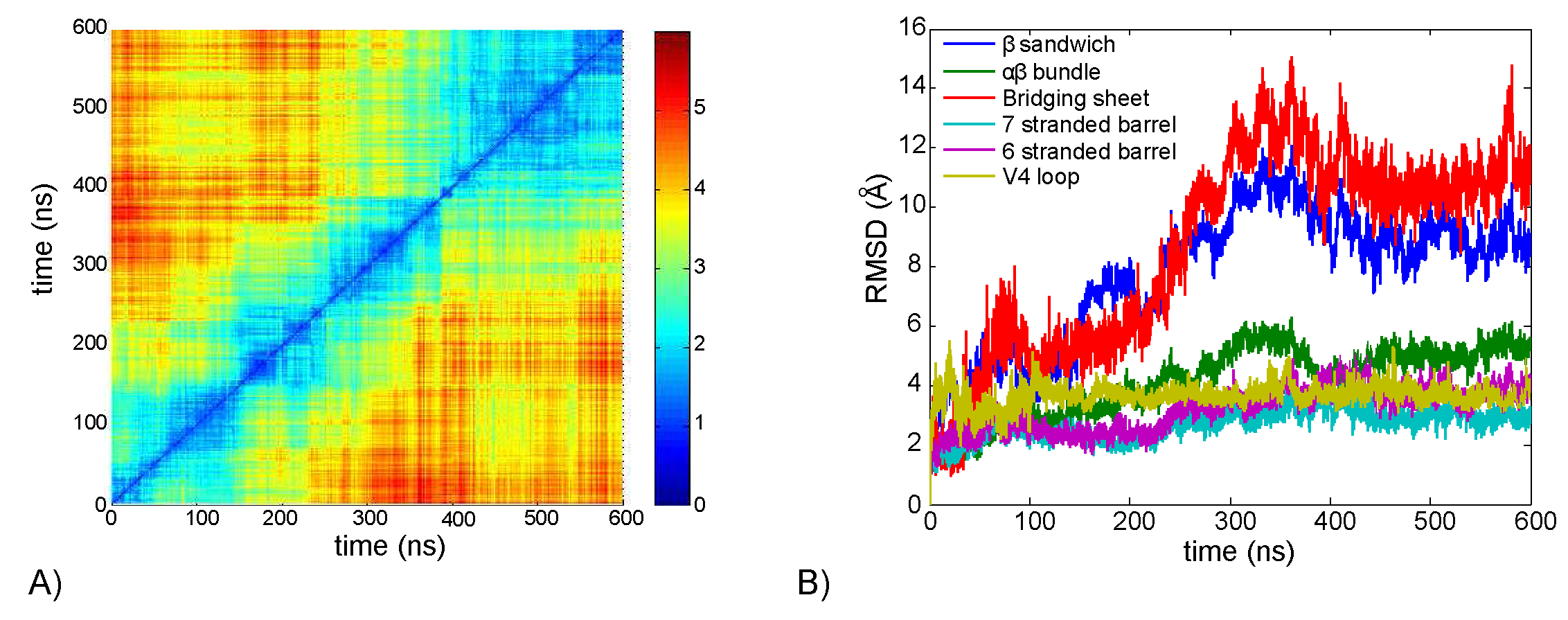

Supplement: Figure S4 — RMSD of the conformations along the 600 ns trajectory in the YU2 simulation. (A) The plot is colored by the RMSD between any two frames during the simulation. Blue represents conformations with low RMSD (high structural similarity) and red represents conformations with high RMSD (low structural similarity). (B) The RMSD of each subdomain of the protein (as compared to the initial conformation) during the simulation is plotted as a function of time. (TIF) [file pcbi.1003046.s004.tif]

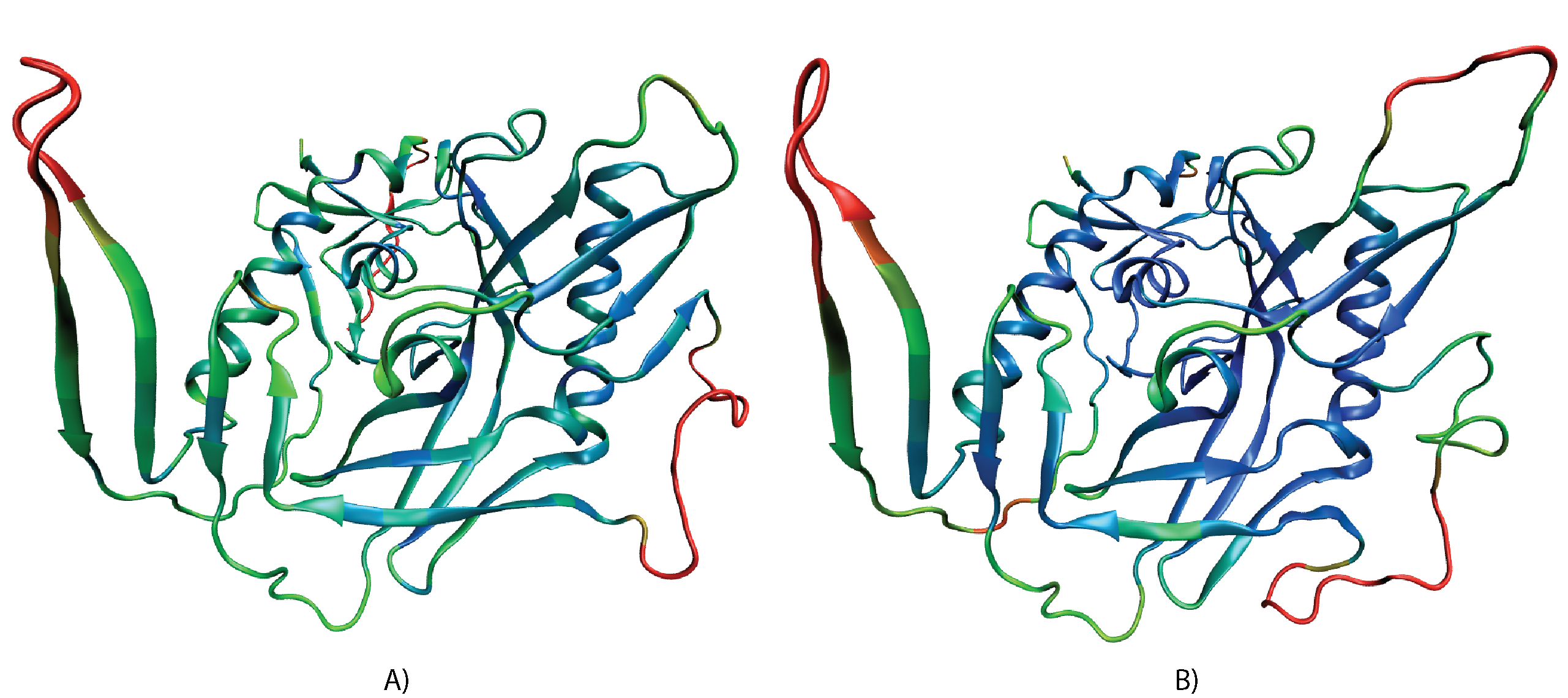

Supplement: Figure S5 — The RMSF per residue during the (A) HXB2 and (B) CAP210 during the 600 ns simulation. (TIF) [file pcbi.1003046.s005.tif]

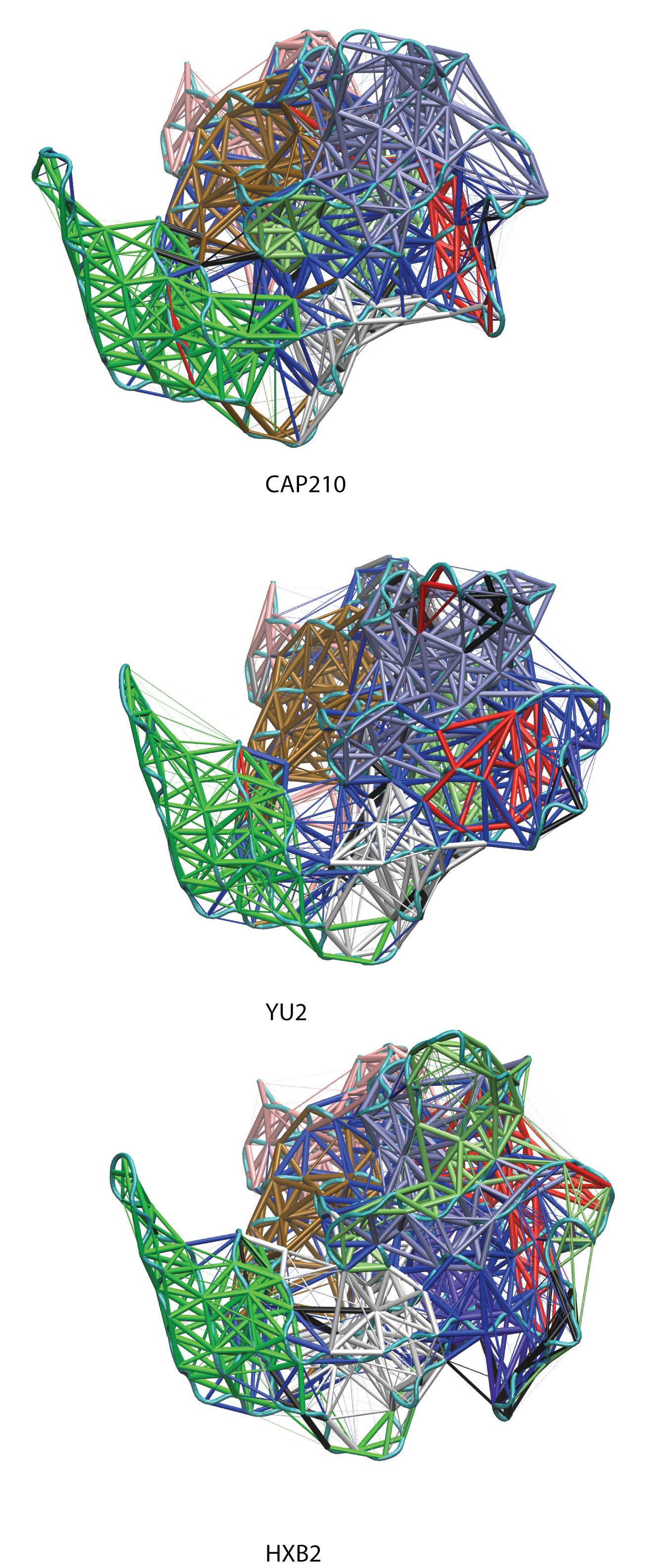

Supplement: Figure S6 — The protein colored by the community each residue belongs to. The backbone of the protein is shown in tube. Again, it would be good to label some of the structures. (TIF) [file pcbi.1003046.s006.tif]

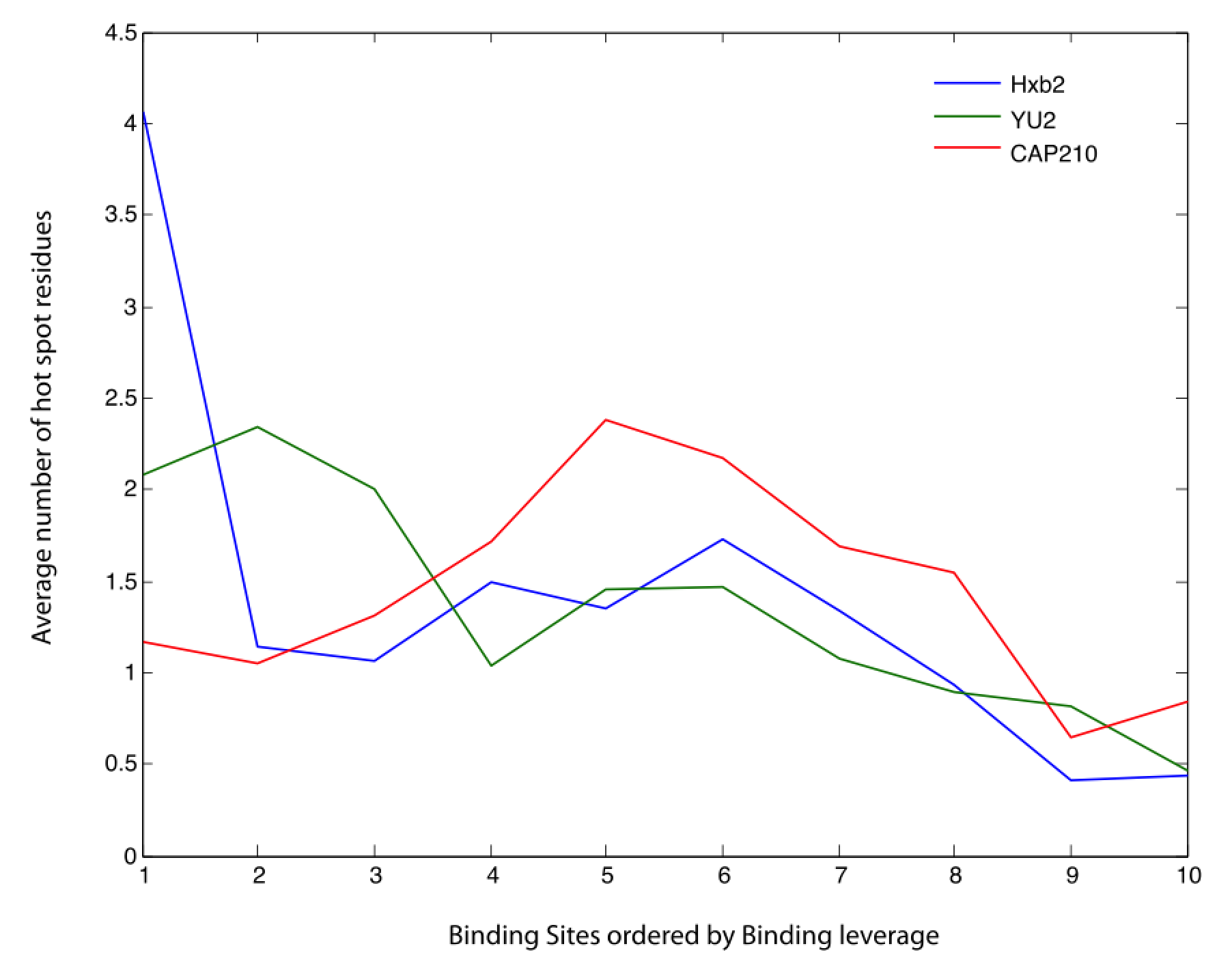

Supplement: Figure S7 — The average number of hotspot residues in 10 blocks of binding sites is plotted. To reduce the noise, the binding sites were arranged by decreasing binding leverage and were divided into 10 blocks. Block 1 contains the sites with the highest binding leverage (10%) while block 10 contains 10% of the sites with the lowest binding leverage. Higher numbers of hotspot residues occur in the sites with highest binding leverage in Hxb2 and YU2 simulations while a higher number of hotspots occur in sites with moderate binding leverage in the CAP210 simulation. In all three simulations, the sites with the lowest binding leverage tend to have a smaller number of hotspot residues. (TIF) [file pcbi.1003046.s007.tif]
