# Supplementary material for: A Mechanistic Understanding of Allosteric Immune Escape Pathways in the HIV-1 Envelope Glycoprotein
Source: PLoS Comput Biol. 2013 May 16;9(5):e1003046. doi: 10.1371/journal.pcbi.1003046 (PMC3656115; doi:10.1371/journal.pcbi.1003046)
Supplement: Table S1 — The overlap of the covariance matrix between different simulations. The values of overlap are between 0.5 and 1.0, indicating that the major coupled motion in the protein is similar for the three different gp120 sequences. (DOCX) [file pcbi.1003046.s008.docx]

|  | **YU2** | **HXB2** | **CAP210** |
| --- | --- | --- | --- |
| **YU2** | **1** | **0.6299** | **0.7236** |
| **HXB2** | **0.6299** | **1** | **0.8734** |
| **CAP210** | **0.7236** | **0.8734** | **1** |
