# Supplementary material for: A Mechanistic Understanding of Allosteric Immune Escape Pathways in the HIV-1 Envelope Glycoprotein
Source: PLoS Comput Biol. 2013 May 16;9(5):e1003046. doi: 10.1371/journal.pcbi.1003046 (PMC3656115; doi:10.1371/journal.pcbi.1003046)
Supplement: Table S2 — Hotspots in YU2, HXB2, and CAP210 networks. (DOCX) [file pcbi.1003046.s009.docx]

| YU2 | HXB2 | CAP210 |
| --- | --- | --- |
| D113 | F93 | D113 |
| K117 | V101 | K117 |
| L122 | D107 | K231 |
| T123 | T110 | S256 |
| V200 | T232 | L260 |
| I201 | F233 | S264 |
| P212 | T236 | A266 |
| P214 | V255 | E268 |
| C228 | Q258 | V275 |
| K232 | L261 | K284 |
| N234 | S264 | S334 |
| G250 | A266 | E335 |
| R252 | V271 | L349 |
| V255 | R273 | N386 |
| Q258 | I284 | S397 |
| L261 | Q287 | D399 |
| I285 | L288 | T410 (insertion in CAP210) |
| Q287 | E293 | N410 (insertion in CAP210) |
| S291 | N295 | T413 |
| C296 | I333 | I414 |
| W338 | R335 | T415 |
| L342 | Q363 | P417 |
| H374 | I371 | R421 |
| F376 | F376 | I423 |
| F382 | N386 | L452 |
| F383 | T388 | L453 |
| W395 | L390 | D457 |
| C417 | I414 | G459 |
| I424 | K421 | F468 |
| W427 | T423 | D477 |
| E429 | I424 | N478 |
| L453 | K432 | S481 |
| T455 | L453 | L483 |
| P469 | R469 |  |
| G471 | G472 |  |
| D477 | W479 |  |
| N478 | R480 |  |
| L483 | R482 |  |
| Y484 | L483 |  |
| K485 | Y484 |  |
|  | K485 |  |
|  | Y486 |  |
