# Supplementary material for: A Mechanistic Understanding of Allosteric Immune Escape Pathways in the HIV-1 Envelope Glycoprotein
Source: PLoS Comput Biol. 2013 May 16;9(5):e1003046. doi: 10.1371/journal.pcbi.1003046 (PMC3656115; doi:10.1371/journal.pcbi.1003046)
Supplement: Table S3 — The hot spot residues in each network that occur at the interface (within 4.5 Angstroms of antibody) of antibody binding sites are listed. The residue number shown in the table refers to the HXB2 sequence numbering while the residue name corresponds to the strain of gp120 in the PDB structure. (DOCX) [file pcbi.1003046.s010.docx]

| **17b (2NY6)** | | **b13 (3IDX)** | | **b12 (2NY7)** | | **F105 ( 3HI1)** | | **VRC01 (3NGB)** | |
| --- | --- | --- | --- | --- | --- | --- | --- | --- | --- |
| L122 | YU2 | I371 | HXB2 | I371 | HXB2 | V255 | HXB2, YU2 | K282 | CAP210 |
| V200 | YU2 | N386 | HXB2, CAP210 | N386 | HXB2, CAP210 | I371 | HXB2 | I371 | HXB2 |
| K421 | HXB2, CAP210 | K421 | HXB2, CAP210 | R421 | HXB2, CAP210 | F376 | HXB2, YU2 | T455 | YU2 |
| I423 | HXB2, CAP210 | D477 | YU2, CAP210 | D477 | YU2, CAP210 | G382 | YU2 | D457 | CAP210 |
|  |  |  |  |  |  | K421 | HXB2, CAP210 | G459 | CAP210 |
|  |  |  |  |  |  | E429 | YU2 | R469 | HXB2, YU2 |
|  |  |  |  |  |  | G472 | HXB2 |  |  |
|  |  |  |  |  |  | D477 | YU2, HXB2, CAP210 |  |  |
