# Supplementary material for: A Mechanistic Understanding of Allosteric Immune Escape Pathways in the HIV-1 Envelope Glycoprotein
Source: PLoS Comput Biol. 2013 May 16;9(5):e1003046. doi: 10.1371/journal.pcbi.1003046 (PMC3656115; doi:10.1371/journal.pcbi.1003046)
Supplement: Table S4 — Binding Leverage of hot spot residues identified using community analysis from YU2 simulation. The binding leverage of a residue refers to the highest binding leverage of a site in which the hotspot residue is present. (DOCX) [file pcbi.1003046.s011.docx]

| **Residue** | **Binding Leverage** |
| --- | --- |
| V255 | 12606 |
| W427 | 12606 |
| K117 | 12606 |
| I285 | 12480 |
| T455 | 12480 |
| G471 | 12480 |
| P212 | 5457 |
| P214 | 5457 |
| D477 | 5394 |
| D113 | 4511 |
| C296 | 3561 |
| T123 | 2755 |
| I424 | 2440 |
| F382 | 2440 |
| R252 | 1838 |
| F376 | 1701 |
| L122 | 1215 |
| Y484 | 951 |
| I201 | 750 |
| E429 | 729 |
| L261 | 690 |
| H374 | 690 |
| Q258 | 690 |
| C417 | 687 |
| L453 | 532 |
| C228 | 469 |
| P469 | 267 |
| L483 | 260 |
| K485 | 260 |
| F383 | 183 |
| S291 | 176 |
| K232 | 166 |
| Q287 | 153 |
| N234 | 146 |
| G250 | 53 |
| V200 | 50 |
| W395 | 24 |
| L342 | 14 |
| W338 | 14 |
| N478 | 1 |
