# Supplementary material for: A Mechanistic Understanding of Allosteric Immune Escape Pathways in the HIV-1 Envelope Glycoprotein
Source: PLoS Comput Biol. 2013 May 16;9(5):e1003046. doi: 10.1371/journal.pcbi.1003046 (PMC3656115; doi:10.1371/journal.pcbi.1003046)
Supplement: Table S5 — Binding Leverage of hotspot residues identified using community analysis from CAP210 simulation. The binding leverage of a residue refers to the highest binding leverage of a site in which the hotspot residue is present. (DOCX) [file pcbi.1003046.s012.docx]

| **Residue** | **Binding Leverage** |
| --- | --- |
| D477 | 7347 |
| N478 | 5153 |
| S481 | 5153 |
| L483 | 3898 |
| E268 | 3898 |
| K231 | 2817 |
| K117 | 2570 |
| L260 | 2179 |
| L452 | 2179 |
| T388 | 2089 |
| D113 | 2026 |
| V275 | 1860 |
| K284 | 1860 |
| I423 | 1294 |
| T410 (insertion in CAP210) | 1210 |
| P417 | 891 |
| S256 | 878 |
| N386 | 656 |
| S334 | 331 |
| R421 | 195 |
| T415 | 195 |
| N410 (insertion in CAP210) | 133 |
| D457 | 113 |
| L349 | 113 |
| L453 | 99 |
| F468 | 95 |
| T413 | 56 |
| E335 | 56 |
| I414 | 56 |
| S264 | 11 |
| G459 | 4 |
| A266 | 4 |
| S397 | 3 |
| D399 | 2 |
