# Supplementary material for: A Mechanistic Understanding of Allosteric Immune Escape Pathways in the HIV-1 Envelope Glycoprotein
Source: PLoS Comput Biol. 2013 May 16;9(5):e1003046. doi: 10.1371/journal.pcbi.1003046 (PMC3656115; doi:10.1371/journal.pcbi.1003046)
Supplement: Table S6 — Binding Leverage of hotspot residues identified using community analysis from HXB2 simulation. The binding leverage of a residue refers to the highest binding leverage of a site in which the hotspot residue is present. (DOCX) [file pcbi.1003046.s013.docx]

| **Residue** | **Binding Leverage** |
| --- | --- |
| Y486 | 12549 |
| V271 | 12549 |
| K485 | 12549 |
| L483 | 12549 |
| Q287 | 12549 |
| R482 | 12549 |
| A266 | 12549 |
| R273 | 9132 |
| Y484 | 8421 |
| S264 | 8421 |
| R480 | 4097 |
| T236 | 3401 |
| I414 | 3387 |
| Q363 | 2089 |
| T388 | 2089 |
| I424 | 1910 |
| K432 | 1910 |
| T232 | 1835 |
| V255 | 1774 |
| F376 | 1774 |
| Q258 | 1633 |
| L261 | 1633 |
| E293 | 1241 |
| L390 | 897 |
| K421 | 893 |
| L453 | 586 |
| I284 | 586 |
| T423 | 444 |
| L288 | 414 |
| R480 | 413 |
| I371 | 270 |
| R469 | 185 |
| T110 | 185 |
| D107 | 185 |
| G472 | 185 |
| F93 | 177 |
| N386 | 167 |
| I333 | 152 |
| V101 | 141 |
| R335 | 76 |
| N295 | 64 |
| W479 | 49 |
| F233 | 34 |
