# Supplementary material for: A Mechanistic Understanding of Allosteric Immune Escape Pathways in the HIV-1 Envelope Glycoprotein
Source: PLoS Comput Biol. 2013 May 16;9(5):e1003046. doi: 10.1371/journal.pcbi.1003046 (PMC3656115; doi:10.1371/journal.pcbi.1003046)
Supplement: Text S1 — Additional results and methods are presented in Text S1. (DOCX) [file pcbi.1003046.s014.docx]

**Supporting Information:**

**Additional Results**

**Flexibility of gp120 from three different strains**

We initially used graph theory to predict the rigid and flexible regions in monomeric gp120. These predictions were then compared with long timescale MD simulations. The FIRST (Floppy Inclusions and Rigid Substructure Topography) method [[1](#_ENREF_1),[2](#_ENREF_2)] constructs a network based on the covalent and noncovalent interactions between atoms in the protein. The balance between the number of degrees of freedom of each atom and the number of constraints required to satisfy these interactions is used to predict the rigidity/flexibility of each residue. In the case of the gp120 monomer, graph theory predicts that the protein is most flexible near the N- and C-termini, the bridging sheet region, and the variable loops V4-V5 (Figure S3A). The core of the outer domain is predicted to be very rigid while the core of the inner domain is moderately flexible.

We compared the predictions from FIRST with the root mean square fluctuations (RMSF) of each residue in the protein during our all-atom MD simulations (Figure S3B). The trends predicted by the FIRST analysis of the YU2 structure are comparable to the RMSF of different regions in YU2 observed during the MD simulation (Correlation of 0.50) and to the flexibility of the gp120 core measured experimentally using hydrogen/deuterium exchange coupled to mass spectrometry (HD-MS). Similar to HD-MS experiments of deglycosylated gp120 core, the outer domain is more rigid than the inner domain and the CD4 binding loop, while the N- and C-termini, and the stem of the V1V2 loop (part of bridging sheet) are the most flexible regions in the protein. Interestingly, the axis of the α1 helix underwent a small rotation with respect to the outer domain during the MD simulations. The orientation of the inner domain changes with respect to the outer domain as gp120 binds to the CD4 receptor [[3](#_ENREF_3),[4](#_ENREF_4)], indicating that gp120 displays a slight tendency to move away from the liganded conformation.

We then compared the flexibility of the protein from the three different sequences of gp120. The flexibility of different regions in gp120 is very similar in all three simulations (correlation coefficient of 0.65-0.75). In these simulations, YU2 is the most flexible, while the CAP210 simulation is least flexible. While the V1V2 stem and the two termini of the protein are more flexible in the YU2 simulation, the V4 loop is more flexible in the HXB2 simulation, and the V5 loop is more flexible in the CAP210 simulation. This indicates that within the same framework of rigidity and flexibility, subtle changes are present in the dynamics of the protein that are dictated by its sequence.

We also tracked the temporal profile of conformational changes in the protein during MD simulations. In order to monitor large conformational changes, we calculated the frame-to-frame root mean square deviation (RMSD) of the protein during the simulation. A frame was chosen every 0.1 ns of the MD simulation during this analysis. As the largest conformational changes were observed in the YU2 simulation, we discuss this simulation here (Figures S3 to S5). An initial relaxation of the protein is followed by subtle orientation changes of the inner domain with respect to the outer domain during the first 100 ns (Figure S4A). The β1 strand at the N-terminus of the protein breaks away from the rest of the β-sandwich in the inner domain of the protein at around 150 ns (Figure S4B). At around 250 ns, the bridging sheet begins to break with the β3 strand losing secondary structure. The bridging sheet continues to be quite flexible during the entire simulation (Figure S3B). This is followed by subtle changes in the orientation of the inner domain with respect to the outer domain and the breaking up of the β2 strand from β22-β23 towards the end of the simulation. The changes are much more subtle in the two remaining simulations, but the trends are similar across all three simulations. In the CAP210 and HXB2 simulations, we observed that the bridging sheet remains intact for all 600 ns even though the stem of the V1V2 loop and the β1-β2 sheets remain mobile. We did not observe any other major conformational changes during the timescales of our simulation, indicating that apo-gp120 is reasonably stable as proposed in a recent X-ray structure comparative study.

The trends in flexibility of different regions in gp120 in MD simulations and predictions from network theory are very similar for all three proteins. However, within this framework, there are subtle differences in the dynamics of the variable regions of the protein that depend upon its sequence. The flexibility of the protein can be used to gain information about the most likely pathway for conformational changes that are necessary for function [[5](#_ENREF_5)]. Identifying the residues that facilitate these pathways can provide additional targets to induce antibodies that prevent gp120 binding to the CD4 receptor. In addition, antibody binding to the flexible regions of gp120 induces a large loss in entropy, which is compensated enthalpically [[6](#_ENREF_6)].

**Convergence of Dynamics:**

In addition to conformational changes in gp120 during the MD simulations, we studied the dynamics of the protein. To measure the coupled motion between different segments of the protein, we used correlation of motion between residues and the principal component analysis (PCA) to evaluate the independent modes of motion in the protein during the MD simulation. To assess the convergence of the coupled motion observed during the MD simulation and the PCA results derived from them, we calculate the eigenvalue-normalized overlap [[7](#_ENREF_7)] for each simulation (Figure S2).

The overlap of the covariance matrix is defined to be 1 only if the two matrices are identical and 0 if the two matrices are completely orthogonal. It has been adopted in a number of studies [[8](#_ENREF_8),[9](#_ENREF_9),[10](#_ENREF_10)] to measure the convergence of a simulation, even though it is not an exact measure of convergence. We compared the covariance matrices calculated over different intervals of the MD simulations with its full duration. The overlap measures the overlap of the phase space visited during different intervals of the simulation. The overlap between the full trajectory and parts of the trajectory increases with the portion of the trajectory used in the calculation. The overlap reaches a value of 0.76-0.89 in the simulations of gp120 started from the CD4-bound conformation. In general, we observe that the motion of the protein seems to have converged in the 600 ns simulations.

We also compared the covariance matrices obtained for the core in different gp120 simulations (Table S1). We observe that the overlap between different simulations is high indicating that the motion of the core in different simulations is similar. The YU2 simulation diverges the most from the other two CD4 bound simulations, as parts of the inner domain started to unfold in this simulation. In addition, it is interesting to note that the dynamics in the CD4 unbound simulation of YU2 displays a high amount of similarity to the dynamics of the protein in the CD4 bound conformation. As both these conformations are highly diverse (especially in the inner domain), it indicates that both conformations have similar types of motion and regions of flexibility in the protein.

**Additional Methods:**

**FIRST Analysis:**

FIRST identifies flexible and rigid regions in three-dimensional bond networks [[1](#_ENREF_1),[2](#_ENREF_2),[5](#_ENREF_5)]. Using this method, the covalent and noncovalent interactions between atoms were initially identified. The nodes in the network represent the atoms while edges represent constraints due to these interactions. The dihedral rotations of bonds are unconstrained and lead to flexibility in a region of the protein if the number of degrees of freedom are greater than the number of constraints (under constrained). A region is considered to be under strain or rigid if the number of constrains are greater than the number of degrees of freedom (over constrained) or all degrees of freedom are associated with exactly the right number of constraints. We applied the FIRST analysis in the FLEXWEB (<http://flexweb.asu.edu/software/first/first_online_newsim/>) website to the model of the core of YU2 with an energy cutoff of -1 kcal/mol for noncovalent interactions.

**Root Mean Square Fluctuation:**

The root mean square fluctuation (RMSF) per residue is a measure of the fluctuation of the position of the residue during the simulation from its mean position. The RMSF is measured over the 600 ns production run for each simulation.  The protein was aligned in VMD [[11](#_ENREF_11)] to remove the translational and rotational motion of the protein using the Kabsch algorithm [[12](#_ENREF_12)] before the RMSF was calculated using the formula:

$$RMSF_{i}=\sqrt{\frac{1}{N}\left(\text{r}_{\text{i}}(t)-\left\langle\text{r}_{\text{i}}(t)\right\rangle\right)^{2}}$$

where **r_i_**(*t*) denotes the position vector of the Cα atom of residue *i* at time *t* and N denotes the total number of frames analyzed and <…> is used to denote the mean of the quantity within the brackets*.*

**References:**

**1. Jacobs DJ, Rader AJ, Kuhn LA, Thorpe MF (2001) Protein flexibility predictions using graph theory. Proteins 44: 150-165.**

**2. Thorpe MF, Lei M, Rader AJ, Jacobs DJ, Kuhn LA (2001) Protein flexibility and dynamics using constraint theory. Journal of molecular graphics & modelling 19: 60-69.**

**3. Diskin R, Marcovecchio PM, Bjorkman PJ (2010) Structure of a clade C HIV-1 gp120 bound to CD4 and CD4-induced antibody reveals anti-CD4 polyreactivity. Nature structural & molecular biology 17: 608-613.**

**4. Abrams CF, Vanden-Eijnden E (2010) Large-scale conformational sampling of proteins using temperature-accelerated molecular dynamics. Proceedings of the National Academy of Sciences of the United States of America 107: 4961-4966.**

**5. Farrell DW, Speranskiy K, Thorpe MF (2010) Generating stereochemically acceptable protein pathways. Proteins 78: 2908-2921.**

**6. Kwong PD, Doyle ML, Casper DJ, Cicala C, Leavitt SA, et al. (2002) HIV-1 evades antibody-mediated neutralization through conformational masking of receptor-binding sites. Nature 420: 678-682.**

**7. Hess B (2002) Convergence of sampling in protein simulations. Physical review E, Statistical, nonlinear, and soft matter physics 65: 031910.**

**8. Grossfield A, Feller SE, Pitman MC (2007) Convergence of molecular dynamics simulations of membrane proteins. Proteins 67: 31-40.**

**9. Lou H, Cukier RI (2006) Molecular dynamics of apo-adenylate kinase: a principal component analysis. The journal of physical chemistry B 110: 12796-12808.**

**10. Cascella M, Micheletti C, Rothlisberger U, Carloni P (2005) Evolutionarily conserved functional mechanics across pepsin-like and retroviral aspartic proteases. Journal of the American Chemical Society 127: 3734-3742.**

**11. Humphrey W, Dalke A, Schulten K (1996) VMD: visual molecular dynamics. Journal of molecular graphics 14: 33-38, 27-38.**

**12. Kabsch W (1978) A discussion of the solution for the best rotation to relate two sets of vectors. Acta Crystallographica Section A 34: 827-828.**
